# Supplementary material for: Neonatal outcome in 29 pregnant women with COVID-19: A retrospective study in Wuhan, China
Source: PLoS Med. 2020 Jul 28;17(7):e1003195. doi: 10.1371/journal.pmed.1003195 (PMC7386573; doi:10.1371/journal.pmed.1003195)
Supplement: S1 STROBE Checklist — STROBE, Strengthening the Reporting of Observational Studies in Epidemiology. (DOCX) [file pmed.1003195.s001.docx]

STROBE Statement—checklist of items that should be included in reports of observational studies

|  | Item No. | Recommendation | Page  No. | Relevant text from manuscript |
| --- | --- | --- | --- | --- |
| **Title and abstract** | 1 | (*a*) Indicate the study’s design with a commonly used term in the title or the abstract | Abstract, Methods and findings | *Retrospective study* |
|  |  | (*b*) Provide in the abstract an informative and balanced summary of what was done and what was found | Abstract, Methods and findings | *Methods and findings* |
| Introduction | | | |  |
| Background/rationale | 2 | Explain the scientific background and rationale for the investigation being reported | Introduction, paragraph 1 | *According to the Chinese Center for Disease Control and Prevention (China CDC), 25.1% of the 44,672 confirmed cases in mainland China were women of reproductive age [3, 4]. Pregnant women experience immunologic and physiologic changes, which make them potentially more susceptible to viral respiratory infections, including respiratory syncytial virus, influenza virus, and SARS-CoV [5]. There is growing evidence that COVID-19 infections in pregnant women are not more severe than in age-matched women where symptoms are typically mild [6-12], and childbirth did not aggravate the course of the illness or chest computed tomography (CT) features of COVID-19 [13]. In addition, previous research suggests infants under 1 year old are susceptible to COVID-19 when in contact with infected family members [14]. However, for neonates born to infected women, little is known about their susceptibility.* |
| Objectives | 3 | State specific objectives, including any prespecified hypotheses | Introduction, paragraph 2 | *In this retrospective study, we analyzed maternal and neonatal clinical characteristics of all women with confirmed or clinical diagnosed COVID-19 infection who gave birth in 2 of the 5 general hospitals in Wuhan, China and followed up their neonates through telephone interview or collecting their clinical data in the only designated children’s hospital to assess neonatal outcomes associated with maternal COVID-19 infection.* |
| Methods | | | |  |
| Study design | 4 | Present key elements of study design early in the paper | Methods, Study design and participants, paragraph 1 | *Retrospective study* |
| Setting | 5 | Describe the setting, locations, and relevant dates, including periods of recruitment, exposure, follow-up, and data collection | Methods, Study design and participants, paragraph 1 | *All pregnant women diagnosed with COVID-19 who gave birth between January 30 to March 10, 2020 in 2 designated general hospitals in Wuhan, China (Renmin Hospital, Wuhan University, and Central Hospital of Wuhan, Tongji Medical College, Huazhong University of Science and Technology) were included in this retrospective study.* |
| Participants | 6 | (*a*) *Cohort study*—Give the eligibility criteria, and the sources and methods of selection of participants. Describe methods of follow-up  *Case-control study*—Give the eligibility criteria, and the sources and methods of case ascertainment and control selection. Give the rationale for the choice of cases and controls  *Cross-sectional study*—Give the eligibility criteria, and the sources and methods of selection of participants | Methods, Maternal data, paragraph 1 | *We followed the diagnostic criteria for COVID-19 in pregnant women according to the New Coronavirus Pneumonia Prevention and Control Program (fifth, sixth, and seventh edition) [15-17] published by the National Health Commission of China (specific diagnostic criteria were shown in S1 Text).* |
|  |  | (*b*) *Cohort study*—For matched studies, give matching criteria and number of exposed and unexposed  *Case-control study*—For matched studies, give matching criteria and the number of controls per case | N/A |  |
| Variables | 7 | Clearly define all outcomes, exposures, predictors, potential confounders, and effect modifiers. Give diagnostic criteria, if applicable | Methods, Neonatal data, paragraph 2; S1 Text 2.4 | *The diagnosis of pneumonia in neonates was based on risk factors, clinical manifestations, radiological findings, and laboratory tests [18]. The diagnosis of COVID-19 in neonates was according to an experts′ consensus statement [19] (specific diagnostic criteria are shown in S1 Text).* |
| Data sources/ measurement | 8* | For each variable of interest, give sources of data and details of methods of assessment (measurement). Describe comparability of assessment methods if there is more than one group | Methods, Maternal data, Neonatal data, and SARS-CoV-2 testing | *Clinical characteristics, examination results, and treatment course were extracted from their medical records.* |
| Bias | 9 | Describe any efforts to address potential sources of bias | Methods, Neonatal data, paragraph 1 | *When parents did not opt for quarantined observation, neonates were discharged after birth. For these neonates, the outcome was obtained through parental telephone interview.* |
| Study size | 10 | Explain how the study size was arrived at | Methods, Study design and participants, paragraph 1 | *All pregnant women diagnosed with COVID-19 who gave birth between January 30 to March 10, 2020 in 2 designated general hospitals in Wuhan, China (Renmin Hospital, Wuhan University, and Central Hospital of Wuhan, Tongji Medical College, Huazhong University of Science and Technology) were included in this retrospective study.* |

Continued on next page

| Quantitative variables | 11 | Explain how quantitative variables were handled in the analyses. If applicable, describe which groupings were chosen and why | Methods, Statistical analysis | *Continuous variables were described as mean (standard deviation [SD]) or median (interquartile range [IQR]). Categorical variables were represented as frequencies with proportions.* |
| --- | --- | --- | --- | --- |
| Statistical methods | 12 | (*a*) Describe all statistical methods, including those used to control for confounding | N/A |  |
|  |  | (*b*) Describe any methods used to examine subgroups and interactions | N/A |  |
|  |  | (*c*) Explain how missing data were addressed | N/A |  |
|  |  | (*d*) *Cohort study*—If applicable, explain how loss to follow-up was addressed  *Case-control study*—If applicable, explain how matching of cases and controls was addressed  *Cross-sectional study*—If applicable, describe analytical methods taking account of sampling strategy | Methods, Neonatal data, paragraph 1 | *When parents did not opt for quarantined observation, neonates were discharged after birth. For these neonates, the outcome was obtained through parental telephone interview.* |
|  |  | (*e*) Describe any sensitivity analyses | N/A |  |
| Results | | | | |
| Participants | 13* | (a) Report numbers of individuals at each stage of study—eg numbers potentially eligible, examined for eligibility, confirmed eligible, included in the study, completing follow-up, and analysed | Fig 1 | *Of the 30 neonates born (including 1 set of twins), 18 were admitted to the Children’s Hospital to have further examination (5 with symptoms and 13 just for quarantine). The other 12 neonates discharged immediately did not undergo imaging screening, and follow-up showed no symptoms or abnormal findings. Among 18 neonates admitted to hospital, 17 neonates had pneumonia-like imaging changes. Five neonates were diagnosed with a COVID-19 infection, including 2 confirmed and 3 suspected.* |
|  |  | (b) Give reasons for non-participation at each stage | Methods, Neonatal data, paragraph 1 | *When parents did not opt for quarantined observation, neonates were discharged after birth. For these neonates, the outcome was obtained through parental telephone interview.* |
|  |  | (c) Consider use of a flow diagram | Fig 1 |  |
| Descriptive data | 14* | (a) Give characteristics of study participants (eg demographic, clinical, social) and information on exposures and potential confounders | Table 1, 2, 3, 4, 5, S1 Table |  |
|  |  | (b) Indicate number of participants with missing data for each variable of interest | N/A |  |
|  |  | (c) *Cohort study*—Summarise follow-up time (eg, average and total amount) | Fig 1 | *The other 12 neonates discharged immediately did not undergo imaging screening, and follow-up showed no symptoms or abnormal findings.* |
| Outcome data | 15* | *Cohort study*—Report numbers of outcome events or summary measures over time | Fig 1 | *Among 18 neonates admitted to hospital, 17 neonates had pneumonia-like imaging changes. Five neonates were diagnosed with a COVID-19 infection, including 2 confirmed and 3 suspected.* |
|  |  | *Case-control study—*Report numbers in each exposure category, or summary measures of exposure | N/A |  |
|  |  | *Cross-sectional study—*Report numbers of outcome events or summary measures | N/A |  |
| Main results | 16 | (*a*) Give unadjusted estimates and, if applicable, confounder-adjusted estimates and their precision (eg, 95% confidence interval). Make clear which confounders were adjusted for and why they were included | N/A |  |
|  |  | (*b*) Report category boundaries when continuous variables were categorized | N/A |  |
|  |  | (*c*) If relevant, consider translating estimates of relative risk into absolute risk for a meaningful time period | N/A |  |

Continued on next page

| Other analyses | 17 | Report other analyses done—eg analyses of subgroups and interactions, and sensitivity analyses | N/A |  |
| --- | --- | --- | --- | --- |
| Discussion | | | | |
| Key results | 18 | Summarise key results with reference to study objectives | Discussion, Main findings | *In this study, among 29 women with confirmed or clinical diagnosed COVID-19 infection, 5 neonates were diagnosed with a COVID-19 infection (2 confirmed and 3 suspected).* |
| Limitations | 19 | Discuss limitations of the study, taking into account sources of potential bias or imprecision. Discuss both direction and magnitude of any potential bias | Discussion, Strengths and limitations | *However, in the early stage of the outbreak, there was a shortage of COVID-19 test kits. As such, most neonates were only tested twice with throat and anal swabs. Only 4 neonates were tested for SARS-CoV-2 specific IgM and IgG. Ai and colleagues [28] reported chest CT had higher sensitivity in diagnosing a COVID-19 infection in comparison to qRT-PCR tests from swab samples in China. Although more than half of the pregnant women in this study were clinically diagnosed based on chest CT images due to the lack of PCR kits, we believe the diagnosis was accurate. Another limitation is the lack of tracheal aspirate to identify specific pathogens from the first 12 neonates with radiological features for pneumonia. This led to the uncertain etiology of the identified pneumonia. Because we found only 5 neonates with (suspected) COVID-19 infection, comparison between groups had limited statistical power.* |
| Interpretation | 20 | Give a cautious overall interpretation of results considering objectives, limitations, multiplicity of analyses, results from similar studies, and other relevant evidence | Conclusions | *Conclusions*  *Neonates born to COVID-19-infected women had an increased risk of COVID-19 and radiological features for pneumonia. Intrapartum and postpartum exposure to their mother may have played a role and intrauterine transmission should not be dismissed. Serum test of SARS-CoV-2 specific IgM and IgG is a valuable test to compensate for a false PCR result. The increased occurrence of necrotizing enterocolitis among neonates born to COVID-19-infected women warrants further investigation.* |
| Generalisability | 21 | Discuss the generalisability (external validity) of the study results | Discussion, Results in context; Discussion, Research implications, paragraph 5 | *Several case series have been reported focusing on the impact of COVID-19 on pregnant women and neonates [6-12]. Chen and colleagues [6] first evaluated the clinical characteristics of COVID-19 infection in 9 mother–neonate pairs. Amniotic fluid, cord blood, neonatal throat swab, and breastmilk samples were tested from 6 patients for SARS-CoV-2, and all were negative. In these case series or case reports, the majority COVID-19-infected pregnant women had mild symptoms and were delivered by CS. Further, all women had lung radiological changes, which is consistent with our results. Although in the study by Zhu and colleagues, 10 neonates experienced several clinical problems including premature labor, shortness of breath, thrombocytopenia, and even death [9]; in other reports, positive neonatal outcomes were minimal. Only 2 cases of confirmed neonatal COVID-19 infection have been reported [24]. One neonate was diagnosed at 17 days postdelivery after close contact with a COVID-19-infected mother and babysitter [25]. The other neonate was found to be infected 36 hours after a CS delivery after negative cord blood, amniotic fluid, and placenta SARS-CoV-2 RNA results [26].*  *Similar findings were found among the 5 infants born to infected women during the severe acute respiratory syndrome (SARS) outbreak [40]. One of the 5 infants born to SARS-infected women developed neonatal respiratory distress syndrome (NRDS) and jejunal perforation, and another developed NEC with ileal perforation shortly after birth. These 2 cases included extremely premature infants in which one was born at 26 weeks’ gestation, and the other was born at 28 weeks. The occurrence of NEC in this study is concerning and warrants further investigation.* |
| Other information | |  | | |
| Funding | 22 | Give the source of funding and the role of the funders for the present study and, if applicable, for the original study on which the present article is based | Funding | *The funders had no role in study design, data collection and analysis, decision to publish, or preparation of the manuscript.* |

*Give information separately for cases and controls in case-control studies and, if applicable, for exposed and unexposed groups in cohort and cross-sectional studies.
