# Supplementary material for: Neonatal outcome in 29 pregnant women with COVID-19: A retrospective study in Wuhan, China
Source: PLoS Med. 2020 Jul 28;17(7):e1003195. doi: 10.1371/journal.pmed.1003195 (PMC7386573; doi:10.1371/journal.pmed.1003195)
Supplement: S1 Text — (DOCX) [file pmed.1003195.s002.docx]

**Statistical analysis plan**

**S1 Text. Statistical Analysis Plan for Project Titled “Neonatal Outcome in 29 Pregnant Women with COVID-19: A Retrospective Study”**

**This file contains:**

1. Our preliminary analysis plan as submitted to the ethical committee (March 10, 2020).

2. The final statistical analysis (April 1, 2020 updated).

**Key Staff:**

Chen Zhang

Andrew Kawai

1. **Original analysis plan**
   1. **Background**

Since December 2019, a novel coronavirus, named severe acute respiratory syndrome coronavirus 2 (SARS-CoV-2), has spread throughout China with human-to-human transmission, and the epidemic has received widespread attention [1]. The virus inevitably continued to spread nearly 100 countries and regions through international routes, and WHO identified the outbreak of Corona Virus Disease 2019 (COVID-19, the disease caused by SARS-CoV-2) as a Public Health Emergency of International Concern (PHEIC) [2]. Although the epidemiological characteristics and pathogenicity of COVID-19 are gradually being revealed, the relationship between coronavirus infection and pregnancy is still limited. According to the report of Chinese Center for Disease Control and Prevention (China CDC), among the 44,672 confirmed cases in mainland China [3, 4], 25.1% were women of reproductive age. Compared with severe acute respiratory syndrome (SARS) and Middle East respiratory syndrome (MERS), which caused by the same coronavirus family, no higher fatality rate was found in COVID-19 (2.9% in Hubei Province, 0.4% outside Hubei Province) but with larger number of infection cases. Pregnant women may be susceptible to pneumonia and have a faster disease deterioration rate due to altered hormone levels and reduced lung volume [5]. Pregnant women experience immunologic tolerance and physiologic changes which might make them more susceptible to viral respiratory infections, including COVID-19. Therefore, it is a critical concern whether COVID-19 will cause severe maternal or neonatal adverse outcomes.

There are currently three case series studies focusing on COVID-19 and pregnancy. Chen et al. [6] reported data describing 9 pregnant women in Wuhan, Hubei Province, who were admitted with COVID-19 between 36 weeks' and 39 weeks plus 4 days' gestation. In another study, Zhu et al. [7] retrospectively analyzed the data of 10 neonates born to 9 mothers with confirmed COVID-19 infection at the gestational age of 31 to 39 weeks. Liu et al. [8] described the clinical course and outcomes of three pregnant women who infected with COVID-19 in their late pregnancy. These cases shown comparable clinical characteristics between pregnant and non-pregnant women with confirmed COVID-19 infection. But the outcome of newborns in Zhu's report is not so optimistic. Several problems were observed in those 10 neonates, such as premature labor (n=6), shortness of breath (n=6), thrombocytopenia accompanied with abnormal liver function (n=2), and even death (n=1). In addition, no evidence supported vertical maternal-fetal transmission by testing the presence of the SARS-CoV-2 in amniotic fluid, umbilical cord blood, neonatal throat swab and breast milk. And a study indicated a very low expression level of angiotensin-converting enzyme 2 (ACE2), the receptor of SARS-CoV-2, by analyzing single-cell profiles in human early maternal–fetal interface [9]. However, as the women at the time of infection are all in their third trimester, and the sample size is still limited, it is too early to draw a conclusion [10]. Therefore, it is necessary to establish a cohort study to collect detailed information of pregnant women with COVID-19 and their neonates, to analyze the impact of COVID-19 on neonates comprehensively.

Reference:

1. Zhu N, Zhang D, Wang W, Li X, Yang B, Song J, et al. A Novel Coronavirus from Patients with Pneumonia in China, 2019. New England Journal of Medicine. 2020;382(8):727-33.
2. WHO Health Alert for coronavirus launches on WhatsApp [Available from: <https://www.who.int/emergencies/diseases/novel-coronavirus-2019/events-as-they-happen>.
3. Novel Coronavirus Pneumonia Emergency Response Epidemiology T. [The epidemiological characteristics of an outbreak of 2019 novel coronavirus diseases (COVID-19) in China]. Zhonghua Liu Xing Bing Xue Za Zhi. 2020;41(2):145-51.
4. Wu Z, McGoogan JM. Characteristics of and Important Lessons From the Coronavirus Disease 2019 (COVID-19) Outbreak in China: Summary of a Report of 72314 Cases From the Chinese Center for Disease Control and Prevention. JAMA. 2020.
5. Yudin MH, Steele DM, Sgro MD, Read SE, Kopplin P, Gough KA. Severe acute respiratory syndrome in pregnancy. Obstet Gynecol. 2005;105(1):124‐127.
6. Chen H, Guo J, Wang C, Luo F, Yu X, Zhang W, et al. Clinical characteristics and intrauterine vertical transmission potential of COVID-19 infection in nine pregnant women: a retrospective review of medical records. The Lancet. 2020;395(10226):809-15.
7. Zhu H, Wang L, Fang C, Peng S, Zhang L, Chang G, et al. Clinical analysis of 10 neonates born to mothers with 2019-nCoV pneumonia. Translational Pediatrics. 2020;9(1):51-60.
8. Coronavirus disease 2019 (COVID-19) during pregnancy: a case series [updated 25 February 2020. Available from: <https://www.preprints.org/manuscript/202002.0373/v1>.
9. Zheng QL, Duan T, Jin LP. Single-cell RNA expression profiling of ACE2 and AXL in the human maternal–Fetal interface. Reprod Dev Med 2020;4:7-10
10. Qiao J. What are the risks of COVID-19 infection in pregnant women? The Lancet. 2020;395(10226):760-2.
    1. **Objective**

To study the clinical outcomes of neonates born to women with COVID-19 infection.

- 1. **Design**

Retrospective cohort study.

- 1. **Setting**

Two designated general hospitals and one designated children’s hospital in Wuhan, China.

There were five designated general hospitals and only one designated children’s hospital in Wuhan. We chose two of the designated general hospitals to establish the cohort.

- 1. **Population**

All the pregnant women with COVID-19 infection who gave birth between January and March 2020 in the two designated general hospitals were included. Their neonates were also included in the study.

- 1. **Main outcome measures**

Neonates infected with COVID-19 or not and its risk factors.

- 1. **Data collection**
     1. **Maternal data**

Sociodemographic data: age, body mass index, educational attainment, occupation, parity;

COVID-19 related medical history: admission date, symptom (fever, cough, shortness of breath, diarrhea, vomiting and so on), chest computed tomography (CT) image, throat swab test, diagnosis, treatment, discharge date;

Laboratory tests pre- and postpartum: routine blood tests (white blood cell count [WBC], lymphocyte count [LYM], lymphocyte percentage [LYM%]), levels of C-reactive protein (CRP) in serum, biochemical indicators of hepatic and renal function (aspartate transaminase [AST], alanine aminotransferase [ALT], creatine kinase [CK], lactate dehydrogenase [LDH], total protein, albumin, uric acid, creatinine, and urea nitrogen) and postnatal blood routine tests and levels of CRP;

Pregnancy outcomes: pregnancy-related complications (gestational hypertensive disorder, gestational diabetes mellitus, premature rupture of membranes, gestational anemia, fetal distress, postpartum hemorrhage), gestational age at delivery, mode of delivery.

- - 1. **Neonatal data**

Basic demographic data: sex, birthweight, 1- and 5-minute Apgar scores, congenital anomalies;

COVID-19 related medical history: admission date, symptoms, Chest X-ray or CT image, throat and anal swab test, diagnosis, treatment, discharge date;

Laboratory tests: routine blood tests (WBC, LYM, LYM%, neutrophil count [NEU], neutrophil percentage [NEU%], platelet count [PLT]), levels of CRP, procalcitonin (PCT) in serum, and biochemical indicators of hepatic and renal function (AST, ALT, CK, LDH, total protein, albumin, uric acid, creatinine, and urea nitrogen).

- 1. **Analytic datasets**

This project aimed to analyze the characteristics and risk factors of neonates infected with COVID-19. This will require analytic datasets: All mothers, Mothers whose child with COVID-19 and Mothers whose child without COVID-19; All neonates, Neonates with COVID-19 and Neonates without COVID-19.

- 1. **Statistical analysis**
     1. Continuous variables: presented as mean/standard deviation (SD) or median/interquartile range (IQR), according to whether they were normally distributed. Normally distributed were analyzed using the unpaired t-test, while not normally distributed data were compared using the Mann-Whitney U-test.
     2. Categorical variables: presented as frequencies with proportions and differences examined using Chi-square test.
     3. All statistical tests were two-tailed, and p-values < 0.05 were considered statistically significant.

Association between neonatal outcome and maternal characteristics: univariate logistic regression analysis was first performed. Multivariate logistic regression was then performed to adjust for multiple potential confounders. Results of the regression models were reported as odds ratios (ORs) with 95% CI.

- 1. **Data representation**

Table 1 Demographic characteristics, pregnancy-related complications, pre- and post-partum conditions and laboratory tests of mothers infected with COVID-19

|  | **All mothers** | **Mothers whose child with COVID-19** | **Mothers whose child without COVID-19** | ***p* value** |
| --- | --- | --- | --- | --- |
|  | **(*n* = )** | **(*n* = )** | **(*n* = )** |  |
|  | **No. (%)** | **No. (%)** | **No. (%)** |  |
| **Age, mean (SD), year** |  |  |  |  |
| **BMI, mean (SD), kg/m^2^** |  |  |  |  |
| **Educational attainment** |  |  |  |  |
| Higher education |  |  |  |  |
| Secondary education |  |  |  |  |
| **Occupation** |  |  |  |  |
| Employed but not at a hospital |  |  |  |  |
| Employed specifically at a hospital |  |  |  |  |
| Unemployed |  |  |  |  |
| **Parity** |  |  |  |  |
| Primiparous |  |  |  |  |
| Multiparous |  |  |  |  |
| **First signs and symptoms** |  |  |  |  |
| Fever |  |  |  |  |
| Cough |  |  |  |  |
| Shortness of breath |  |  |  |  |
| Diarrhea |  |  |  |  |
| Vomiting |  |  |  |  |
| None |  |  |  |  |
| **Throat swab** |  |  |  |  |
| **Chest CT** |  |  |  |  |
| **Prepartum laboratory tests** |  |  |  |  |
| White blood cell count, median (IQR), ×10^9^/L |  |  |  |  |
| Lymphocyte count, median (IQR), ×10^9^/L |  |  |  |  |
| Lymphocyte percentage, median (IQR), % |  |  |  |  |
| C-reactive protein, median (IQR), mg/L |  |  |  |  |
| Aspartate transaminase, median (IQR), U/L |  |  |  |  |
| Alanine aminotransferase, median (IQR), U/L |  |  |  |  |
| Creatine kinase, median (IQR), U/L |  |  |  |  |
| Lactate dehydrogenase, median (IQR), U/L |  |  |  |  |
| Total protein, median (IQR), g/L |  |  |  |  |
| Albumin, median (IQR), g/L |  |  |  |  |
| Uric acid, median (IQR), μmol/L |  |  |  |  |
| Creatinine, median (IQR), μmol/L |  |  |  |  |
| Urea nitrogen, median (IQR), mmol/L |  |  |  |  |
| **Pregnancy-related complications** |  |  |  |  |
| Gestational hypertensive disorder |  |  |  |  |
| Gestational diabetes mellitus |  |  |  |  |
| Gestational anemia |  |  |  |  |
| Preterm premature rupture of membranes |  |  |  |  |
| Fetal distress |  |  |  |  |
| Postpartum hemorrhage |  |  |  |  |
| Other complications |  |  |  |  |
| **Mode of delivery** |  |  |  |  |
| Vaginal |  |  |  |  |
| Cesarean section |  |  |  |  |
| **Number of fetus** |  |  |  |  |
| Singleton |  |  |  |  |
| Twin |  |  |  |  |
| **Postpartum laboratory tests** |  |  |  |  |
| White blood cell count, median (IQR), ×10^9^/L |  |  |  |  |
| Lymphocyte count, median (IQR), ×10^9^/L |  |  |  |  |
| Lymphocyte percentage, median (IQR), % |  |  |  |  |
| C-reactive protein, median (IQR), mg/L |  |  |  |  |

Table 2 Characteristics of neonates born to mothers infected with COVID-19

|  | **All neonates** | **Neonates with COVID-19** | **Neonates without COVID-19** | ***p* value** |
| --- | --- | --- | --- | --- |
|  | **(*n* = )** | **(*n* = )** | **(*n* = )** |  |
|  | **No. (%)** | **No. (%)** | **No. (%)** |  |
| **Sex** |  |  |  |  |
| Male |  |  |  |  |
| Female |  |  |  |  |
| **Gestational age, weeks** |  |  |  |  |
| <37 |  |  |  |  |
| ≥37 |  |  |  |  |
| **Birthweight, g** |  |  |  |  |
| <2500 |  |  |  |  |
| 2500-4000 |  |  |  |  |
| >4000 |  |  |  |  |
| **AGA** |  |  |  |  |
| SGA |  |  |  |  |
| AGA |  |  |  |  |
| LGA |  |  |  |  |
| **Apgar score at 1-min** |  |  |  |  |
| <7 |  |  |  |  |
| 7-10 |  |  |  |  |
| **Apgar score at 5-min** |  |  |  |  |
| <7 |  |  |  |  |
| 7-10 |  |  |  |  |
| **Congenital anomaly** |  |  |  |  |
| **Neonatal fever** |  |  |  |  |
| **Neonatal respiratory distress** |  |  |  |  |
| **NICU admission** |  |  |  |  |

Table 3 Univariable and multivariable logistic regression of neonates with or without COVID-19

| **Variable** | **Neonates with COVID-19** | **Neonates without COVID-19** | **Univariable logistic regression analysis** | **Multivariable logistic regression analysis** | ***p* value** |
| --- | --- | --- | --- | --- | --- |
|  | **(*n* = )** | **(*n* = )** | **(Unadjusted OR, 95% CI)** | **(Adjusted OR, 95% CI)** |  |
| **Variable 1** |  |  |  |  |  |
| **Variable 2** |  |  |  |  |  |
| **Variable 3** |  |  |  |  |  |
| **Variable 4** |  |  |  |  |  |
| **Variable 5** |  |  |  |  |  |

Fig 1 Flow chart of participants.

1. **Final statistical analysis**
   1. **Maternal COVID-19 infection diagnosis criteria**

During the study period, three versions of diagnostic criteria of COVID-19 were introduced according to New Coronavirus Pneumonia Prevention and Control Program (5^th^, 6^th^ and 7^th^ edition). What’s more, due to the shortage of PCR kits in Wuhan, many women did not undergo nucleic acid testing, but diagnosed by CT imaging of typical features of viral pneumonia, which was called “clinical diagnosed COVID-19 infection”.

The specific diagnostic criteria were as follows:

1. 5^th^ edition (published on February 8):
   1. Epidemiological histories:
      1. Have a history of travel or residence in Wuhan or surrounding areas, or other communities with reported cases within 14 days before onset；
      2. Have a history of contact with COVID-19 infection (positive nucleic acid test) within 14 days before onset；
      3. Have contacted patients with fever or respiratory symptoms from Wuhan or surrounding areas, or other communities with reported cases within 14 days before onset；
      4. Clustered onset.
   2. Clinical manifestations:
      1. Fever or respiratory symptoms;
      2. Blood routine tests: the number of white blood cells is normal or decreased in the early stage of onset, or the lymphocyte count is reduced；
   3. Chest X-ray or CT images: in the early stage of onset, there were multiple small patch shadows and interstitial changes, and the extrapulmonary zone was more obvious. Then it developed multiple ground-glass opacities and infiltration shadows in the lungs. In severe cases, lung consolidation may occur, and pleural effusions were rare.
   4. Etiology tests:
      1. Positive SARS-CoV-2 RNA in respiratory or blood samples;
      2. Viral gene sequencing of respiratory or blood samples was highly homologous to SARS-CoV-2.

**Suspected infection** was defined as one of the epidemiological histories plus one of the clinical manifestations, or two clinical manifestations without epidemiological history. **Clinical diagnosed infection** was defined as suspected infection plus typical features in chest X-ray or CT images. **Confirmed infection** was defined as suspected or Clinical diagnosed infection plus one of the etiology tests.

1. 6^th^ edition (published on February 18):
   1. Epidemiological histories: the same as 5^th^ edition.
   2. Clinical manifestations: the clinical manifestations plus chest X-ray or CT images in 5^th^ edition.
   3. Etiology tests: the same as 5^th^ edition.

**Suspected infection** was defined as one of the epidemiological histories plus two of the clinical manifestations, or three clinical manifestations without epidemiological history. **Confirmed infection** was defined as suspected infection plus one of the etiology tests.

1. 7^th^ edition (published on March 4):
   1. Epidemiological histories: on the basis of the epidemiological histories of the 6^th^ edition, a clustered onset was defined, that is, two or more cases of fever and/or respiratory symptoms occurred in a small area such as home, office, school, class, etc. within two weeks).
   2. Clinical manifestations: on the basis of the clinical manifestations of the 6^th^ edition, blood routine tests plus the lymphocyte count could be normal.
   3. Etiology tests: on the basis of the etiology tests of the 6^th^ edition, plus a new Serological test: positive SARS-CoV-2 specific IgM or IgG in serum, or SARS-CoV-2 specific IgG changed from negative to positive, or the levels of SARS-CoV-2 specific IgG in recovery period was four times or more higher than it in the acute period.

**Suspected infection** was defined as one of the epidemiological histories plus two of the clinical manifestations, or three clinical manifestations without epidemiological history. **Confirmed infection** was defined as suspected infection plus one of the etiology tests.

- 1. **Neonatal follow-up**

After birth, neonates who have clinical manifestations (5 cases) and whose guardians agreed with a hospitalized quarantine (13 cases) were admitted to Children’s Hospital. For those hospitalized neonates, laboratory tests and chest X-Ray or CT were performed. Other symptom-free neonates were discharged immediately after birth and followed up through telephone.

- 1. **Maternal and neonatal SARS-CoV-2 IgM and IgG tests**

Due to the late arrival of Detection Kit for serum SARS-CoV-2 IgM and IgG, only one mother and four neonates were tested. In addition, because the Detection Kit has not been certificated, and there is no precedent for detection in the serum of neonates, we classified neonates with only antibody positive but nucleic acid negative as suspected infection.

- 1. **Neonatal COVID-19 infection diagnosis criteria**

There were currently no diagnosis criteria for neonatal COVID-19 infections. Therefore, based on experts’ consensus statement of children COVID-19 infection and the characteristics of the neonates, we defined the diagnosis criteria for neonatal COVID-19, including:

1. Epidemiological history: born to a confirmed COVID-19 infected mother;
2. Clinical manifestations:
3. Symptom: fever, cough or other respiratory symptoms, lethargy, poor feeding, poor reflexes, and cannot be explained by other diseases or pathogens;
4. Chest X-ray or CT images: scattered or diffuse ground-glass opacities in unilateral or bilateral lung, may with focal consolidations, mostly in the peripheral lung, and cannot be explained by other diseases or pathogens;
5. Serological test: positive SARS-CoV-2 specific IgM or IgG in serum (because the Detection Kit has not been certificated);
6. Etiology test: positive SARS-CoV-2 RNA in throat or anal swab samples.

**Suspected infection** was defined as epidemiological history plus one of the clinical manifestations. **Confirmed infection** was defined as suspected infection plus etiology test.

- 1. **Grouping and statistical analysis**

In the original analysis plan, we intended to analyze the risk factors for neonatal COVID-19 infection, so, the mothers were divided into two groups based on whether neonatal COVID-19 occurred. However, in the actual process, we found that five of the 18 hospitalized neonates can be diagnosed as COVID-19 after examination, although two of these five were diagnosed by serological tests and one was diagnosed by typical radiological features of COVID-19 infection (these three were classified as suspected patients). 12 of the remaining 13 hospitalized neonates had radiographic changes of pneumonia characteristics, the other one also had abnormal radiological finding. An additional 12 newborns were not examined, although telephone follow-up showed no symptoms (Fig 1). Therefore, mothers were finally divided as three groups: Mothers whose child had COVID-19 (n=5), Mothers whose child had abnormal radiological findings without COVID-19 (n=12) and Mothers whose child discharged after birth (n=12). In addition, due to the limitation of the sample sizes, we gave up doing comparison between groups and only do a descriptive analysis.

In addition, for five COVID-19 neonates (two confirmed and three suspected), we describe the detailed information of them and their mothers according to the timeline, in order to help readers understand the course of the infection.

- 1. **The occurrence of neonatal necrotizing enterocolitis**

In the actual process, three of the 13 hospitalized neonates without COVID-19 developed necrotizing enterocolitis, although none of them were premature, which we did not think of in the original analysis plan. We were very interested in this, so we collected the symptoms and the onset time of these neonates. But because of the small number of samples, we cannot draw a definitive conclusion. Therefore, in this manuscript, we only mentioned it in the discussion section. More research is needed for further analysis.

- 1. **Data representation**

Original Table 1 was split into two tables for the sake of publication.

Table 1 Demographic characteristics, prepartum conditions and laboratory tests of mothers infected with COVID-19

|  | **All mothers** | **Mothers whose child had COVID-19** | **Mothers whose child had abnormal radiological findings without COVID-19** | **Mothers whose child discharged after birth** |
| --- | --- | --- | --- | --- |
|  | **(*n* = )** | **(*n* = )** | **(*n* = )** | **(*n* = )** |
|  | **No. (%)** | **No. (%)** | **No. (%)** | **No. (%)** |
| **Age, mean (SD), year** |  |  |  |  |
| **BMI, mean (SD), kg/m^2^** |  |  |  |  |
| **Educational attainment** |  |  |  |  |
| Higher education |  |  |  |  |
| Secondary education |  |  |  |  |
| **Occupation** |  |  |  |  |
| Employed but not at a hospital |  |  |  |  |
| Employed specifically at a hospital |  |  |  |  |
| Unemployed |  |  |  |  |
| **Parity** |  |  |  |  |
| Primiparous |  |  |  |  |
| Multiparous |  |  |  |  |
| **First signs and symptoms** |  |  |  |  |
| Fever |  |  |  |  |
| Cough |  |  |  |  |
| Shortness of breath |  |  |  |  |
| Diarrhea |  |  |  |  |
| Vomiting |  |  |  |  |
| None |  |  |  |  |
| **Throat swab** |  |  |  |  |
| **Chest CT** |  |  |  |  |
| **Prepartum laboratory tests** |  |  |  |  |
| **White blood cell count, median (IQR), ×10^9^/L** |  |  |  |  |
| <3.5 |  |  |  |  |
| 3.5-9.5 |  |  |  |  |
| >9.5 |  |  |  |  |
| **Lymphocyte count, median (IQR), ×10^9^/L** |  |  |  |  |
| <1.1 |  |  |  |  |
| 1.1-3.2 |  |  |  |  |
| >3.2 |  |  |  |  |
| **Lymphocyte percentage, median (IQR), %** |  |  |  |  |
| **C-reactive protein, median (IQR), mg/L** |  |  |  |  |
| **Aspartate transaminase, median (IQR), U/L** |  |  |  |  |
| **Alanine aminotransferase, median (IQR), U/L** |  |  |  |  |
| **Creatine kinase, median (IQR), U/L** |  |  |  |  |
| **Lactate dehydrogenase, median (IQR), U/L** |  |  |  |  |
| **Total protein, median (IQR), g/L** |  |  |  |  |
| **Albumin, median (IQR), g/L** |  |  |  |  |
| **Uric acid, median (IQR), μmol/L** |  |  |  |  |
| **Creatinine, median (IQR), μmol/L** |  |  |  |  |
| **Urea nitrogen, median (IQR), mmol/L** |  |  |  |  |

Table 2 Pregnancy-related complications, postpartum conditions and laboratory tests of mothers infected with COVID-19

|  | **All mothers** | **Mothers whose child had COVID-19** | **Mothers whose child had abnormal radiological findings without COVID-19** | **Mothers whose child discharged after birth** |
| --- | --- | --- | --- | --- |
|  | **(*n* = )** | **(*n* = )** | **(*n* = )** | **(*n* = )** |
|  | **No. (%)** | **No. (%)** | **No. (%)** | **No. (%)** |
| **Pregnancy-related complications** |  |  |  |  |
| Gestational hypertensive disorder |  |  |  |  |
| Gestational diabetes mellitus |  |  |  |  |
| Gestational anemia |  |  |  |  |
| Preterm premature rupture of membranes |  |  |  |  |
| Fetal distress |  |  |  |  |
| Postpartum hemorrhage |  |  |  |  |
| Other complications |  |  |  |  |
| **Mode of delivery** |  |  |  |  |
| Vaginal |  |  |  |  |
| Cesarean section |  |  |  |  |
| **Number of fetus** |  |  |  |  |
| Singleton |  |  |  |  |
| Twin |  |  |  |  |
| **White blood cell count, median (IQR), ×10^9^/L** |  |  |  |  |
| **Lymphocyte count, median (IQR), ×10^9^/L** |  |  |  |  |
| **Lymphocyte percentage, median (IQR), %** |  |  |  |  |
| **C-reactive protein, median (IQR), mg/L** |  |  |  |  |
| Values are presented as median (interquartile range, IQR), or frequency (proportion). | | | | |

Table 3 Clinical features of mothers whose neonates was infected with COVID-19

|  | **Case No.** | **Case No.** | **… …** | **… …** | **… …** |
| --- | --- | --- | --- | --- | --- |
| **Admission day** |  |  |  |  |  |
| **Symptoms** |  |  |  |  |  |
| **Chest CT** |  |  |  |  |  |
| **Throat swab** |  |  |  |  |  |
| **SARS-CoV-2 IgM, AU/mL** |  |  |  |  |  |
| **SARS-CoV-2 IgG, AU/mL** |  |  |  |  |  |
| **White blood cell count, ×109/L** |  |  |  |  |  |
| **Lymphocyte count, ×109/L** |  |  |  |  |  |
| **Lymphocyte percentage, %** |  |  |  |  |  |
| **C-reactive protein, mg/L** |  |  |  |  |  |
| **Aspartate transaminase, U/L** |  |  |  |  |  |
| **Alanine aminotransferase, U/L** |  |  |  |  |  |
| **Creatine kinase, U/L** |  |  |  |  |  |
| **Lactate dehydrogenase, U/L** |  |  |  |  |  |
| **Total protein, g/L** |  |  |  |  |  |
| **Albumin, g/L** |  |  |  |  |  |
| **Uric acid, μmol/L** |  |  |  |  |  |
| **Creatinine, μmol/L** |  |  |  |  |  |
| **Urea nitrogen, mmol/L** |  |  |  |  |  |
| **Treatment** |  |  |  |  |  |
| **Pregnant-related complications** |  |  |  |  |  |
| **Mode of delivery** |  |  |  |  |  |
| **COVID-19 diagnosis** |  |  |  |  |  |
|  | | | | | |

Table 4 Clinical features of neonates with COVID-19

|  | **Case No.** | **Case No.** | **… …** | **… …** | **… …** |
| --- | --- | --- | --- | --- | --- |
| **Date of birth (Day1)** |  |  |  |  |  |
| **Days from mother diagnosed, days** |  |  |  |  |  |
| **Sex** |  |  |  |  |  |
| **Gestational age, weeks** |  |  |  |  |  |
| **Birthweight, g** |  |  |  |  |  |
| **AGA** |  |  |  |  |  |
| **Apgar score at 1-min** |  |  |  |  |  |
| **Apgar score at 5-min** |  |  |  |  |  |
| **Congenital anomaly** |  |  |  |  |  |
| **Symptoms** |  |  |  |  |  |
| **Throat swab** |  |  |  |  |  |
| **Anal swab** |  |  |  |  |  |
| **Chest X-ray or CT after birth** |  |  |  |  |  |
| **White blood cell count, ×109/L** |  |  |  |  |  |
| **Lymphocyte count, ×109/L** |  |  |  |  |  |
| **Lymphocyte percentage, %** |  |  |  |  |  |
| **C-reactive protein, mg/L** |  |  |  |  |  |
| **Procalcitonin, ng/mL** |  |  |  |  |  |
| **SARS-CoV-2 IgM, AU/mL** |  |  |  |  |  |
| **SARS-CoV-2 IgG, AU/mL** |  |  |  |  |  |
| **Treatment** |  |  |  |  |  |
| **NICU stay, days** |  |  |  |  |  |
| **Hospitalization stay, days** |  |  |  |  |  |
| **X-ray or CT before discharge** |  |  |  |  |  |
| **COVID-19 diagnosis** |  |  |  |  |  |

Table 5. Characteristics of hospitalized neonates without COVID-19

|  | **Case No.** | **Case No.** | **… …** |
| --- | --- | --- | --- |
| **Date of birth** |  |  |  |
| **Days from mother diagnosed, days** |  |  |  |
| **Sex** |  |  |  |
| **Gestational age, weeks** |  |  |  |
| **Birthweight, g** |  |  |  |
| **AGA** |  |  |  |
| **Apgar score at 1-min** |  |  |  |
| **Apgar score at 5-min** |  |  |  |
| **Congenital anomaly** |  |  |  |
| **Fever** |  |  |  |
| **Other symptoms** |  |  |  |
| **Throat swab** |  |  |  |
| **Anal swab** |  |  |  |
| **Chest X-ray or CT after birth** |  |  |  |
| **Treatment** |  |  |  |
| **NICU stay, days** |  |  |  |
| **Hospitalization stay, days** |  |  |  |
| **X-ray or CT before discharge** |  |  |  |
| **Discharge diagnosis** |  |  |  |

Table 6 Laboratory tests of all hospitalized neonates born to mother with COVID-19

|  | **Reference range a** | **Case No.** | **Case No.** | **… …** |
| --- | --- | --- | --- | --- |
| **Date of tests b** |  |  |  |  |
| **White blood cell count, ×10^9^/L** |  |  |  |  |
| **Lymphocyte count, ×10^9^/L** |  |  |  |  |
| **Lymphocyte percentage, %** |  |  |  |  |
| **Neutrophil count, ×109/L** |  |  |  |  |
| **Neutrophil percentage, %** |  |  |  |  |
| **Platelet count, ×109/L** |  |  |  |  |
| **C-reactive protein, mg/L** |  |  |  |  |
| **Procalcitonin, ng/mL** |  |  |  |  |
| **Aspartate transaminase, U/L** |  |  |  |  |
| **Alanine aminotransferase, U/L** |  |  |  |  |
| **Creatine kinase, U/L** |  |  |  |  |
| **Lactate dehydrogenase, U/L** |  |  |  |  |
| **Total protein, g/L** |  |  |  |  |
| **Albumin, g/L** |  |  |  |  |
| **Uric acid, μmol/L** |  |  |  |  |
| **Creatinine, μmol/L** |  |  |  |  |
| **Urea nitrogen, mmol/L** |  |  |  |  |
| **Total IgM, g/L** |  |  |  |  |
| **Total IgG, g/L** |  |  |  |  |
| **SARS-CoV-2 IgM, AU/mL** |  |  |  |  |
|  |  |  |  |  |
| **SARS-CoV-2 IgG, AU/mL** |  |  |  |  |
|  |  |  |  |  |

Fig 1 Flow chart of participants inclusion.

Fig 2 Chest X-ray or computed tomography (CT, transverse plane) images of neonates diagnosed with COVID-19 infection.

Fig 3 Chest X-ray or computed tomography (CT, transverse plane) images of neonates with radiological change in chest but were not diagnosed with COVID-19.
