# Supplementary material for: Neonatal outcome in 29 pregnant women with COVID-19: A retrospective study in Wuhan, China
Source: PLoS Med. 2020 Jul 28;17(7):e1003195. doi: 10.1371/journal.pmed.1003195 (PMC7386573; doi:10.1371/journal.pmed.1003195)
Supplement: S2 Text — COVID-19, coronavirus disease 2019. (DOCX) [file pmed.1003195.s003.docx]

**S2 Text. Brief medical histories of 5 neonates with COVID-19 infection.**

**Abbreviations:** COVID-19, coronavirus disease 2019; CRP, C-reactive protein; CT, computed tomography; GGO, ground-glass opacity; IgG, immunoglobulin G; IgM, immunoglobulin M; LYM, lymphocyte count; LYM%, lymphocyte percentage; NICU, neonatal intensive care unit; PCT, procalcitonin; SARS-CoV-2, severe acute respiratory syndrome coronavirus 2

**Patient 5** was born at 40^+4^ weeks’ gestation and weighed 3360g. The mother was admitted to the hospital for a chest CT examination because of a cough and found typical COVID-19 pneumonia changes. The neonate was delivered by cesarean section that night (Day 1) in a negative-pressure operation room. The next day after birth, she was sent to the children's hospital for further observation. Total IgM, IgG, blood routine, CRP, PCT, biochemical indicators of hepatic and renal functions in serum was detected (Table 4). Chest CT showed GGO in the posterior basal segment of left lung lower lobe (Fig 1A). In the following 8 days, she underwent 4 throat and anal swab SARS-CoV-2 RNA tests, of which the second showed positive (the samples on Day 5). No special medication was provided given the infant had no COVID-19 symptoms. Chest X-ray film before discharge showed no abnormal findings (Fig 1B). Based on the typical CT changes and a positive etiology test, the baby girl was diagnosed as confirmed COVID-19 infection.

**Patient 9** was born at 39^+1^ weeks’ gestation and weighed 3570g. The mother was admitted to hospital due to threatened labor and gestational diabetes mellitus. Although she did not display common COVID-19 infection symptoms, a chest CT revealed focal GGO in bilateral lungs, combined with the results of routine blood (LYM 1.32×10^9^/L, LYM% 9.6%) and CRP levels (16.9mg/L), she was clinically diagnosed with a COVID-19 infection. That evening (Day 1) she had an emergency caesarean section. The next day (Day 2), a weakly positive result was found in the neonate’s throat swab and a chest CT showed pneumonia changes (bilateral diffuse GGO with multifocal consolidations, Fig 1C). The neonate developed a low-grade fever (37.5℃) on Day 3. Although throat swab and anal swab tests were negative on Day 5, the throat swab test was positive on Day 7. A subsequent chest X-ray revealed patchy obscure shadows in bilateral lung lower fields (Fig 1D). Throat swab and anal swab samples were negative on Day 10 and Day 13. After treatment with interferon and Amoxicillin-Clavulanate potassium, chest X-ray on Day 17 showed complete disappearance of pneumonia (Fig 1E). NICU discharge was on Day 21 but within 48 hours the infant developed a fever (38.5℃) and was readmitted. During the second NICU stay, throat and anal swabs were both negative, the levels of specific IgM and IgG to SARS-CoV-2 were negative on Day 26. After symptomatic support treatment, the infant discharged again on Day 31. Combining CT images with the positive throat swab on Day 7, the infant was considered confirmed COVID-19 infected.

**Patient 10** was a female neonate born at 38^+3^ weeks’ gestation (Day 1) with an immediate chest CT image showing characteristic COVID-19 changes of bilateral patchy GGO (Fig 1F). The neonate was transferred to the NICU and underwent a series of tests, all of which were negative including three throat and anal swab tests on Day 2, 5, and 9. Symptomatic supportive treatment was provided and on Day 13, a chest X-ray showed significant release of pneumonia (Fig 1G). A further chest X-ray on Day 20 showed no abnormalities (Fig 1H). The SARS-CoV-2 specific IgM and IgG tested was completed on Day 28 and was positive (IgM, 10.65 AU/mL; IgG, 80.46 AU/mL). She was discharged 2 days later with the diagnosis as suspected COVID-19.

**Patient 12** was a female infant born at 37^+1^ week’s gestation with a birth weight of 2940g. Her mother had a fever for 10 days (maximum body temperature reached 38℃), accompanied by cough and vomiting. She was admitted to the hospital for throat swab test and it showed positive (35^+1^ weeks’ gestation). Due to the good physical foundation, she chose to continue to conceive and receive antiviral treatment. After 14 days, the infant was delivered by cesarean section with atrial septal defect (Day1). Immediately after birth, the infant was sent to the Children's Hospital for chest CT examination, which showed typical viral pneumonia findings. Although three consecutive throat and anal swab tests were all negative (Day 3, Day 4, Day 7), the infant was diagnosed as a suspected COVID-19 case considering that the manifestations of viral pneumonia in imaging could not be explained by other reasons. After interferon inhalation, partial pneumonia lesions were absorbed and she was discharged from NICU to the general isolation ward.

**Patient 18** has been previously described in a case report by Dr. Lan Dong^1^. This female neonate was born to a woman with confirmed COVID-19 infection at 37^+6^ weeks gestation (Day 1) with an immediate chest CT image suggesting possible pneumonia with reduced latency with GGO in bilateral lower lobes (Fig 1I). Upon NICU admission on Day 2, throat swab, anal swab, and laboratory tests were performed. Levels of CRP and PCT were negative at admission and four throat and anal swab tests were negative on Day 3, 6, 10, and 14. On Day 1 and Day 15, SARS-CoV-2 specific IgM and IgG were positive. In addition, chest X-ray and CT images showed typical findings of viral infection: increased bilateral lung markings and increased densities of previous existing pulmonary lesions with interlobular septal thickening (Fig 1J, 1K). Due to the typical findings in chest CT and positive SARA-CoV-2 serum IgM and IgG, this infant was diagnosed as suspected COVID-19 infection.

**Reference**

Dong L, Tian J, He S, Zhu C, Wang J, Liu C, et al. Possible Vertical Transmission of SARS-CoV-2 From an Infected Mother to Her Newborn. JAMA. 2020;323(18):1846-1848. http://doi.org/10.1001/jama.2020.4621 PMID: 32215581; PubMed Central PMCID: PMCPMC7099527.
