# Supplementary material for: Neonatal outcome in 29 pregnant women with COVID-19: A retrospective study in Wuhan, China
Source: PLoS Med. 2020 Jul 28;17(7):e1003195. doi: 10.1371/journal.pmed.1003195 (PMC7386573; doi:10.1371/journal.pmed.1003195)
Supplement: S1 Table — COVID-19, coronavirus disease 2019. (DOCX) [file pmed.1003195.s004.docx]

**S1 Table. Laboratory tests of all hospitalized neonates born to mother with COVID-19.**

| **Patient** | **Ref range ^a^** | **1** | **2** | **3** | **4** | **5** | **6** | **7** | **8** | **9** | **10** | **11** | **12** | **13** | **14** | **15** | **16** | **17** | **18** |
| --- | --- | --- | --- | --- | --- | --- | --- | --- | --- | --- | --- | --- | --- | --- | --- | --- | --- | --- | --- |
| **Date of tests ^b^** |  | Day 2 | Day 4 | Day 2 | Day 2 | Day 2 | Day 2 | Day 2 | Day 2 | Day 5 | Day 1 | Day 1 | Day 1 | Day 4 | Day 1 | Day 2 | Day 2 | Day 2 | Day 3 |
| **White blood cell count, ×10^9^/L** | 5-30 | 18.56 | 10.99 | 15.76 | 16.27 | 19.23 | 24.12 | 21.56 | 20.97 | 14.27 | 16.59 | 22.95 | 18.79 | 8 | 8.77 | 11.61 | 18.02 | 26.63 | 19.29 |
| **Lymphocyte count, ×10^9^/L** | 2-17 | 2.54 | 1.6 | 2.27 | 1.37 | 2.61 | 2.96 | 2.71 | 4.58 | 2.19 | 2.45 | 2.87 | 2.95 | 2.68 | 2.46 | 1.46 | 2.24 | 5.98 | 4.3 |
| **Lymphocyte percentage, %** | 31-43 | 13.7 | 14.6 | 14.4 | 8.5 | 13.6 | 12.3 | 12.6 | 21.8 | 15.3 | 14.8 | 12.5 | 15.7 | 33.5 | 28 | 12.6 | 12.4 | 22.5 | 22.3 |
| **Neutrophil count, ×109/L** | 3.9-9.4 | 14.49 | 7.86 | 11.9 | 13.36 | 15.3 | 18.69 | 17.44 | 14.46 | 9.99 | 12.89 | 17.83 | 13.59 | 3.68 | 4.98 | 9.42 | 14.59 | 17.17 | 13.19 |
| **Neutrophil percentage, %** | 31-52 | 78.1 | 71.5 | 75.5 | 82.1 | 79.5 | 77.5 | 81 | 69 | 70 | 77.7 | 77.7 | 73.9 | 45.9 | 56.7 | 81.1 | 81.1 | 64.4 | 68.4 |
| **Platelet count, ×109/L** | 242-378 | 254 | 276 | 341 | 347 | 265 | 314 | 272 | 333 | 302 | 367 | 340 | 240 | 336 | 340 | 319 | 307 | 394 | 281 |
| **C-reactive protein, mg/L** | 0-3 | <0.75 | <0.75 | <0.75 | <0.75 | <0.75 | 0.97 | 1.32 | <0.75 | 11.4 | <0.75 | <0.75 | 1.09 | 0.96 | <0.75 | <0.75 | <0.75 | <0.75 | <0.75 |
| **Procalcitonin, ng/mL** | 0-0.5 | (-) | (-) | (-) | (-) | (-) | 12 | NA | NA | 2.95 | NA | 2.48 | NA | NA | 1.88 | NA | NA | NA | NA |
| **Aspartate transaminase, U/L** | 15-46 | 43 | 81 | 44 | 66 | 103 | 99 | 67 | 156 | 30 | 72 | 197 | 39 | 85 | 94 | 37 | 47 | 49 | 102 |
| **Alanine aminotransferase, U/L** | 21-72 | 30 | 35 | 20 | 24 | 47 | 29 | 19 | 32 | 25 | 20 | 28 | 22 | 22 | 27 | 19 | 21 | 19 | 35 |
| **Creatine kinase, U/L** | 30-170 | 324 | 660 | 385 | 583 | 985 | 851 | 689 | 358 | 474 | 458 | 742 | 905 | 1085 | 829 | 421 | 357 | 737 | 967 |
| **Lactate dehydrogenase, U/L** | 192.5-735 | 446 | 635 | 426 | 724 | 533 | >753 | >753 | >753 | 355 | 359 | >753 | 325 | 630 | 370 | 352 | 502 | 358 | 621 |
| **Total protein, g/L** | 63-82 | 60.2 | 56.9 | 60.8 | 55.6 | 59.7 | 65.6 | 59.5 | 66.1 | 61.3 | 67.6 | 64 | 56.2 | 66 | 61.4 | 57.5 | 59.6 | 59.6 | 55.6 |
| **Albumin, g/L** | 35-50 | 34.4 | 33.2 | 35.5 | 34.2 | 34.9 | 37.6 | 35.6 | 39.8 | 34.6 | 39.5 | 39.3 | 31.4 | 38.8 | 34.2 | 31.2 | 34.8 | 34.2 | 34.4 |
| **Uric acid, μmol/L** | 208-506 | 224 | 343.1 | 382.6 | 433.4 | 357.7 | 405.5 | 199.6 | 351.5 | 184.1 | 330.3 | 185.2 | 238.2 | 179.5 | 330.7 | 373.5 | 335.5 | 317.8 | 355.7 |
| **Creatinine, μmol/L** | 58-110 | 69 | 52 | 53.2 | 80.4 | 66.8 | 79.1 | 69.4 | 60.9 | 50.1 | 57.3 | 97.3 | 50.5 | 46 | 55.1 | 53.9 | 46.6 | 55.2 | 76.1 |
| **Urea nitrogen, mmol/L** | 3.2-7.1 | 5.59 | 5.38 | 1.92 | 6.94 | 2.87 | 3.2 | 5.64 | 3.61 | 2.12 | 1.37 | 3.39 | 1.33 | 1.11 | 2.38 | 2.62 | 2.72 | 2.62 | 7.94 |
| **Total IgM, g/L** | 0.05-0.3 | 0.18 | 0.18 | 0.18 | NA | <0.18 | 0.18 | 0.18 | 0.18 | <0.18 | NA | NA | 0.18 | 0.19 | <0.18 | <0.18 | <0.18 | 0.18 | 0.18 |
| **Total IgG, g/L** | 7-14.8 | 6.7 | 7.8 | 7.46 | NA | 8.75 | 11.4 | 9.63 | 7.98 | 8.73 | NA | NA | 7.9 | 9.32 | 9.27 | 9.65 | 8.97 | 9.06 | 5.89 |
| **SARS-CoV-2 IgM, AU/mL** | 0-10 | NA | NA | NA | NA | NA | NA | NA | NA | 0.46 (Day26) | 10.65  (Day28) | 0.77 (Day 25) | NA | NA | NA | NA | NA | NA | 45.83 (Day 1); 11.75 (Day 15) |
| **SARS-CoV-2 IgG, AU/mL** | 0-10 | NA | NA | NA | NA | NA | NA | NA | NA | 2.36 (Day 26) | 80.46  (Day28) | 33.92 (Day 25) | NA | NA | NA | NA | NA | NA | 140.32 (Day1); 69.94 (Day15) |

^a^ The reference range changes depending on the age of the neonates.

^b^ “Date of tests” refers to the days of life when the test is performed.

SI conversion factors: To convert aspartate transaminase, alanine aminotransferase, creatine kinase and lactate dehydrogenase to μkat/L, multiply values by 0.0167.

COVID-19, coronavirus disease 2019; IgG, immunoglobulin G; IgM, immunoglobulin M; NA, not available; SARS-CoV-2, severe acute respiratory syndrome coronavirus 2.
