# Supplementary figures and images for: Neonatal outcome in 29 pregnant women with COVID-19: A retrospective study in Wuhan, China
Source: PLoS Med. 2020 Jul 28;17(7):e1003195. doi: 10.1371/journal.pmed.1003195 (PMC7386573; doi:10.1371/journal.pmed.1003195)

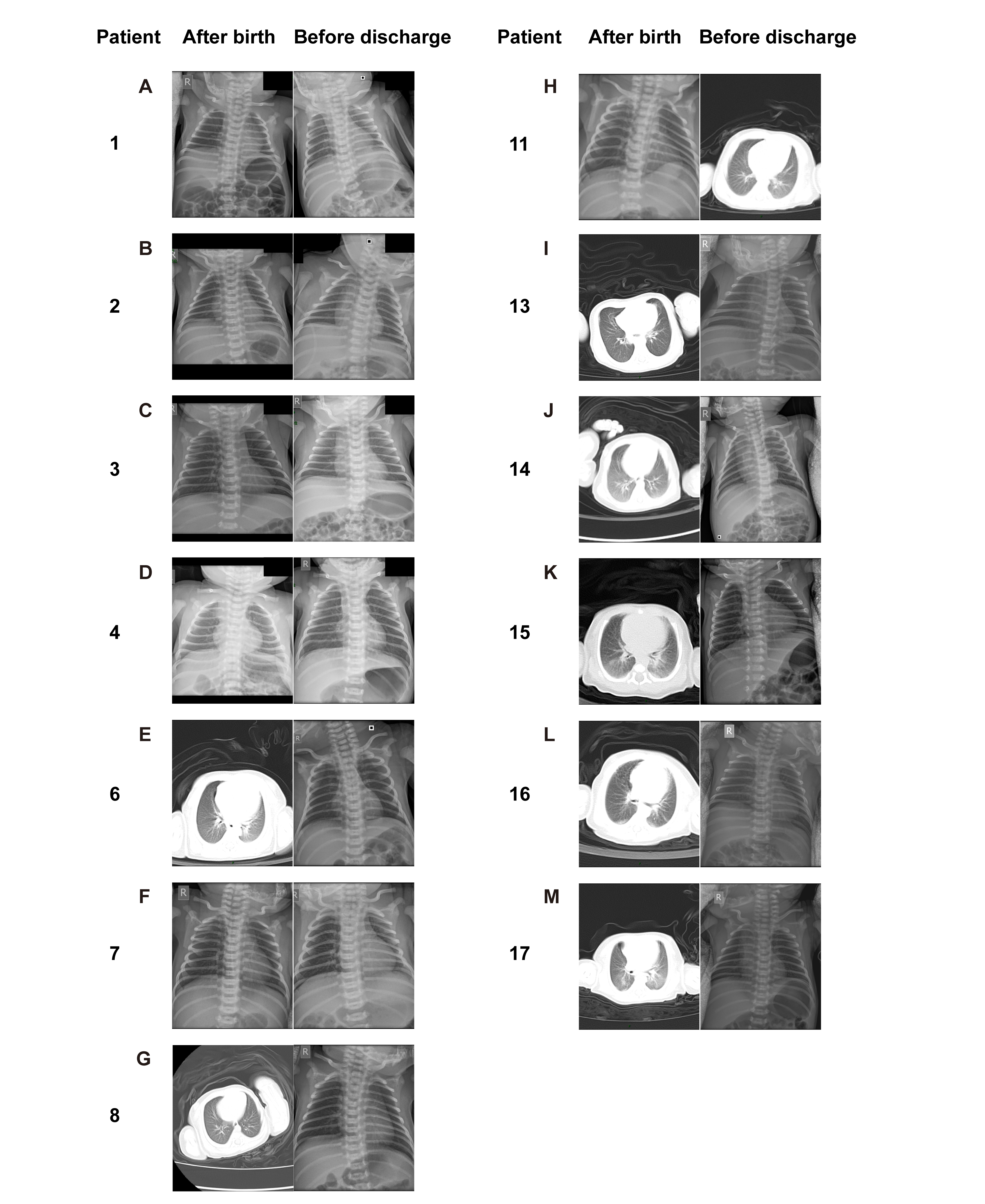

Supplement: S1 Fig — Left panel of each case was the chest X-ray or CT performed after birth (within 3 days, mostly within 24 hours). Except one showing increased bilateral lung markings (H, Patient 11), the other 12 showed the typical manifestations of pneumonia. The main findings was diffuse or scattered patchy obscure shadows of unilateral or bilateral lungs, 5 cases with partial ground-glass opacities (G, I, J, K, M, Patient 8, 13, 14, 15, 17), 5 cases with peripheral focal consolidations (E, I, K, L, M, Patient 6, 13, 15, 16, 17), and one case with paramediastinal emphysema (E, Patient 6). Right panel of each case was the chest X-ray performed before discharge (Patient 11 was performed after discharge), of which 12 neonates with pneumonia-like radiological changes showed absorption of lesions. Four cases showed slightly increasing lung markings (B, C, D, F, Patient 2, 3, 4, 7). Only one case showed scattered patchy blurry shadows (K, Patient 15). The other one still showed increased bilateral lung markings (H, Patient 11). All images have been de-identified to protect patient privacy. COVID-19, coronavirus disease 2019; CT, computed tomography. (TIF) [file pmed.1003195.s005.tif]

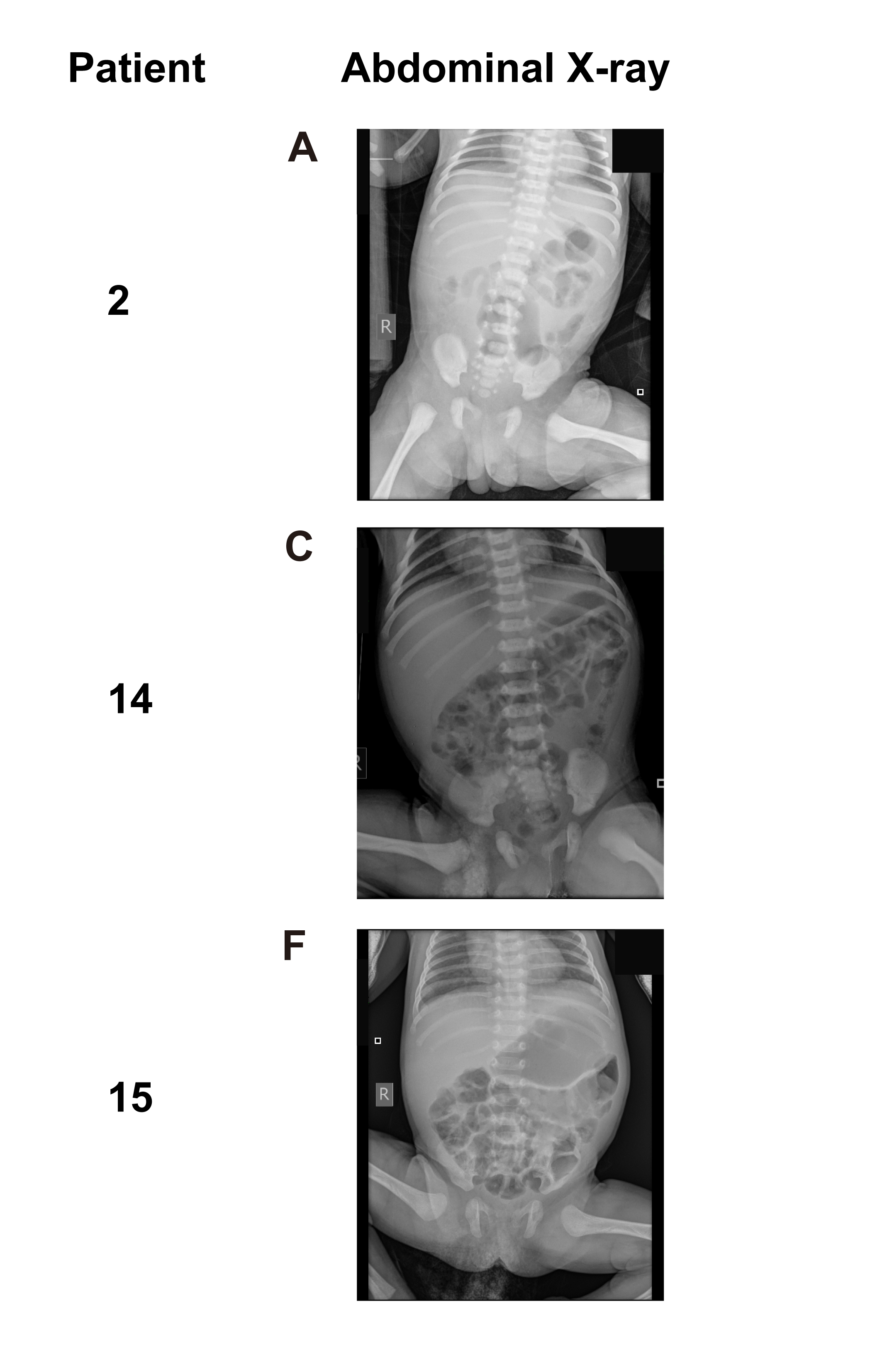

Supplement: S2 Fig — (A, Patient 2) Abdominal X-ray images at Day 5 showed intermediate abdominal pneumatosis, indistinct intestinal space between bowels in the middle and lower abdomen, and the presence of “bubble sign”. (B, Patient 14) Abdominal X-ray images at Day 5 showed abdominal pneumatosis; indistinct intestinal space between bowels in the right lower abdomen, and slightly linear and bubble-like radiolucencies. (C, Patient 15) Abdominal X-ray images at Day 20 showed mild abdominal pneumatosis, which was mainly located in the colon, and slightly indistinct intestinal space between bowels in the left lower abdomen. All images have been de-identified to protect patient privacy. (TIF) [file pmed.1003195.s006.tif]
