# Supplementary material for: Neonatal outcome in 29 pregnant women with COVID-19: A retrospective study in Wuhan, China
Source: PLoS Med. 2020 Jul 28;17(7):e1003195. doi: 10.1371/journal.pmed.1003195 (PMC7386573; doi:10.1371/journal.pmed.1003195)
Supplement: Attachment — Submitted filename: Response to Reviewers.docx [file pmed.1003195.s007.docx]

**Comments from the editor:**

1. *Thank you for noting your intent to share the data. At this time, please provide the link to the data repository and accession numbers required for access. If you intend to include the data as a supplementary file, please include the data files with your revision and reference the supplemental file from the main text (in the Methods section).*

**Response:** This has been adjusted as requested. All the research data was available at the ResMen Manager of Chinese Clinical Trial Registry (www.medresman.org), and the registration number is ChiCTR2000031954 (http://www.medresman.org.cn/pub/cn/proj/projectshshow.aspx?proj=1810).

(Please see Page 10, Line 202-204)

2. *Thank you for providing your STROBE statement. Please replace the page numbers with paragraph numbers per section (e.g. "Methods, paragraph 1"), since the page numbers of the final published paper may be different from the page numbers in the current manuscript.*

**Response:** Thank you for your suggestion. A new STROBE statement has been uploaded.

(Please see S1 Checklist)

3. *If a prospective analysis plan (from your funding proposal, IRB or other ethics committee submission, study protocol, or other planning document written before analyzing the data) was used in designing the study, please include the relevant prospectively written document with your revised manuscript as a supplemental file. If no such document currently exists, please provide a supplemental file detailing the original analysis plan. The analysis plan and your methods should clearly state (1) the specific hypotheses you intended to test, (2) the analytical methods by which you planned to test them, (3) the analyses you actually performed, and (4) when reported analyses differ from those that were planned, provide transparent explanations for differences that affect the reliability of the study's results. For example, if a reported analysis was performed based on an interesting but unanticipated pattern in the data, please be clear that the analysis was data-driven. If hypotheses that were not included in the original study design later became important to test because new evidence became available from other studies, please explain the situation, so that it is clear whether new analyses were data driven or added for another reason. Please see our guidelines for observational studies here: http://journals.plos.org/plosmedicine/s/submission-guidelines#loc-guidelines-for-specific-study-types*

**Response:** As mentioned, although this is a retrospective study, we had a preliminary analysis plan when applying for ethical review and clinical trial registration. However, during the actual data collection process we adjusted the grouping and statistical analysis methods. All these modifications are described in the uploaded new S1 Text.

(Please see S1 Text)

4. *Thank you for noting Dr. Mol’s competing interests. Please clarify the last sentence “No potential conflicts of interest.” If it is meant to refer to the grant and consultancies reported by Dr. Mol, this sentence can be rephrased to say “These consultancies are not related to the current work” or similar. If it is meant to refer to the other authors, please rephrase to say “All other authors report no potential competing interests.” or similar. Either way, please do confirm whether any other authors have relevant competing interests.*

**Response:** We have now adjusted the statement to increase clarity and it now reads as follows: “Ben W. Mol is supported by…These consultancies are not related to the current work. All other authors report no potential competing interests.”

(Please see Page 20, Line 439-443)

5. *Please revise your title according to PLOS Medicine's style. Please place the study design ("A randomized controlled trial," "A retrospective study," "A modelling study," etc.) in the subtitle (ie, after a colon).*

**Response:** We have modified the title as requested and now reads: “Neonatal Outcome in 29 Pregnant Women with COVID-19: A Retrospective Study”.

(Please see Page 1, Line 1)

6. *The entire manuscript would benefit from a thorough check for grammar, spelling, and clarity.*

**Response:** Thank you for your suggestion. The manuscript has now been reviewed by two English-speaking professors.

7. *In the last sentence of the Abstract Methods and Findings section, please describe the main limitation(s) of the study's methodology.*

**Response:** As requested, we have now added the following sentence: “The limited sample size limited statistical comparison between groups.”

(Please see Page 5, Line 99)

8. *Abstract Conclusions: Please be more specific, and address the study implications without overreaching what can be concluded from the data; the phrase "In this study, we observed ..." may be useful.*

**Response:** We have modified the text as follows: “In this study, we observed 17% (5/30) neonates born to COVID-19 infected women had COVID-19 and 57% (17/30) had radiological features of pneumonia.”

(Please see Page 5, Line 100-102)

9. *The Introduction could benefit with more background on what is known and not known about COVID-19 in pregnant women, infants, and other populations. Additionally, a bit more on the setting and population of the current study would be useful.*

**Response:** This has been modified as requested.

(Please see Page 6, Line 119-125, 126-130)

10. *Please include the Ethical approval and Informed consent sections in the Methods.*

**Response:** This study was reviewed by the medical ethics committees in the three participating hospitals. We have now added this to the study design section and have removed it from the end of manuscript.

(Please see Page 7, Line 140-147)

11. *Results lines 197-198: please present numerators and denominators for percentages.*

**Response:** This has been modified as requested.

(Please see Page 10, Line 210-213)

12. *Line 206: “uneventful follow-up” could be more specific.*

**Response:** This has been modified as requested. The new sentence reads as follows: “The 12 neonates discharged immediately did not undergo imaging screening and followed up showed no symptoms or abnormal findings.”

(Please see Page 10, Line 220-221)

13. *Please present and organize the Discussion as follows: a short, clear summary of the article's findings; what the study adds to existing research and where and why the results may differ from previous research; strengths and limitations of the study; implications and next steps for research, clinical practice, and/or public policy; one-paragraph conclusion.*

**Response:** This has been modified as requested. We now use these subheadings to distinguish the key sections of the discussion: Main findings; Results in context; Strengths and limitations; Research implications.

(Please see Page 13-18, Line 276-387)

14. *Please use the "Vancouver" style for reference formatting, and present reference numbers in square brackets. Please see our website for other reference guidelines https://journals.plos.org/plosmedicine/s/submission-guidelines#loc-references*

**Response:** This has been modified as requested.

**Comments from the reviewers:**

***Reviewer #1****: I confine my remarks to statistical aspects of this paper.*

*These were very simple but appropriately so and I recommend publication*

*Peter Flom*

**Response:** Thank you for agreement on this work.

***Reviewer #2****: The article indeed offer a series of relatively complete data,including Maternal demographic characteristics, delivery course, symptoms, and laboratory tests. However some researchs about the outcome of neonates born to infected women have published, the innovation of the paper needs to be improved. Suggest to transfer to Letter format.*

**Response:** Although there are more and more case series reported focusing on the impact of COVID-19 on pregnant women and neonates, none of these studies have integrated the complete data of mothers and neonates for analysis. Our data comes from two of the five designated general hospitals in Wuhan, and the only designated children's hospital. All data are from primary sources, which has eliminated the recall bias often found in retrospective studies. Importantly, our study clearly indicates that neonates born to COVID-19 infected women had an increased risk of COVID-19 infection and radiological features of pneumonia. Further, due to the continuous follow-up, we identified the occurrence of necrotizing enterocolitis in 3 full-term neonates. All of these findings are not reported in the other studies.

***Reviewer #3:*** *REVIEWER COMMENTS TO AUTHOR*

*Thank you for the opportunity to review this paper.*

*There is no doubt that the COVID-19 pandemic will be of interest to PLOS Medicine's wide general audience. I believe the content of this manuscript will lead to a substantial advance in clinical management of pregnant women (and their neonates) with COVID-19. Further, the data presented provide a substantial new insight into the pathogenesis of COVID-19. I support the publication of this manuscript.*

*General comments, strengths and weaknesses*

*I believe the authors have done a good job pulling together disparate data from two hospitals with multiple clinicians involved. Whilst I think the 17% vertical/neonatal transmission is interesting, the radiologic changes of pneumonia in the non-COVID neonates are remarkable. I think the authors should make more of this unique finding. What does it mean to have 12/30 neonates from COVID mothers with radiologic pneumonia in relatively well neonates? What is the cause of the radiologic changes? What are the potential diagnoses responsible for these radiologic changes?*

**Response:** Thank you for this constructive comment. In our study, 12/13 hospitalized neonates without COVID-19 infection had radiologic pneumonia and only one had an occasional cough. We think the reasons behind this could be one of the following:

1. We noticed that maternal antenatal CRP levels were higher in those with a neonate who had an abnormal radiological finding: in the mothers of the five children with COVID-19, median CRP was 18.65 mg/L, versus 12.20 mg/L in mothers of the other 12 children with radiological abnormalities and 1 mg/L in the 12 children with an uneventful clinical course. The antenatal CRP levels may reflect the maternal inflammation state which impacts the fetal immune state. (Please see Page 15, Line 313-319)
2. Several neonates may be infected with other pathogenic microorganisms as indicated by the elevated CRP or PCT levels. Because none of the neonates had symptoms, no other microbiological test was conducted. (Please see Page 16, Line 352-354)
3. Due to the false positives of the nucleic acid detection kit and the inability to test the same asymptomatic neonates repeatedly, some of these neonates with radiologic pneumonia changes may have had COVID-19 infection. (Please see Page 16, Line 354-359)
4. Finally, all these neonates were delivered by cesarean section so none of them experienced the squeeze of the birth canal. A small amount of amniotic fluid will have inevitably remained in their respiratory tract, which may have caused similar manifestations in lung imaging. (Please see Page 16, Line 359-360). In addition, after symptomatic treatment the lung lesions were all absorbed, so in the end these neonates were not diagnosed with pneumonia.

*Specific points:*

*1. What did the researchers do and find?*

*a. Line 51 - while the other 12 neonates WERE discharged after birth ….*

**Response:** This has been corrected.

(Please see Page 3, Line 52)

*b. Line 53 - It would be easier to understand if you wrote 'Five of the 18 hospitalized neonates…'*

**Response:** This has been corrected.

(Please see Page 3, Line 54-55)

*c. I think you should also put in a comment about the NEC in the COVID negative neonates who were not preterm.*

**Response:** The comment has been added: “	Three of the 13 other hospitalized neonates developed necrotizing enterocolitis, although none of them were premature.”

(Please see Page 3, Line 60-61)

*2. What do these findings mean?*

*a. I think the vertical transmission and radiologic features of pneumonia should be two separate points. It is interesting that 17% of neonates developed COVID-19. It is remarkable that 12/13 (92%) of the non-COVID neonates had radiologic signs of pneumonia. This should be emphasised.*

**Response:** As requested, the points have been adjusted as follows:

•	17% (5/30) neonates born to COVID-19 infected women developed COVID-19.

•	57% (17/30) neonates born to COVID-19 infected women had radiological feature for pneumonia.”

(Please see Page 3, Line 63-65)

*3. Abstract*

*a. Line 71 - manifestations TO non-pregnant women*

**Response:** This has been corrected.

(Please see Page 4, Line 76)

*b. Lines 88-90 - this sentence does not make sense.*

**Response:** This sentence has been removed.

(Please see Page 5, Line 94-97)

*c. Line 91 - presented WITH radiological features…*

**Response:** This has been corrected.

(Please see Page 5, Line 96)

*d. Line 96 - radiological FEATURES OF pneumonia.*

**Response:** This has been corrected.

(Please see Page 5, Line 102)

*4. Introduction - clear*

*5. Methods*

*a. Line 136 remove WAS*

**Response:** This has been corrected.

(Please see Page 7, Line 149)

*b. Line 144 maternal history should be MATERNAL PARITY*

**Response:** This has been corrected.

(Please see Page 8, Line 157)

*6. Results*

*a. Line 196 - suggest AVAILABLE rather than supply*

**Response:** This has been corrected.

(Please see Page 10, Line 210)

*b. Line 221-222 - S1 Table is really important. I think this should be upgraded to Table 5*

**Response:** Thank you for your suggestion. While we would like to upgrade this supplementary table to Table 5, the large number of cases described (13 cases) make it too large to be displayed within two pages. Thus, this may need to be discussed with the editors.

*c. Line 242 - I think you should add after …abnormal radiological findings… (without diagnosis of COVID-19)*

**Response:** As requested, we have adjusted the sentence as follows: “but three neonates with abnormal radiological findings without COVID-19 infection had neonatal necrotizing enterocolitis (NEC) and were therefore admitted longer.”

(Please see Page 13, Line 267-269)

*7. Discussion*

*a. Line 265-266 - 'In the present study, a detailed item might play a role' does not make sense. I suggest re-writing the sentence.*

**Response:** We have removed this sentence.

(Please see Page 14, Line 289)

*b. Line 289 - 'false negatives or false positives.' I suggest FALSE NEGATIVE OR FALSE POSITIVE RESULTS.*

**Response:** This has been adjusted.

(Please see Page 15, Line 325)

*c. Line 291 - I suggest changing 'proposal' to THE LATE ARRIVAL OF THE TESTING PLATFORM.*

**Response:** This has been adjusted.

(Please see Page 15, Line 326-327)

*d. Lines 312-314. What does the 'pneumonia-like damage' mean to the neonate in the short term and the long term? What do you think they have as their diagnoses?*

**Response:** Thank you for the insightful comments. According to our current follow-up, after evidence-based treatment with antibiotics or symptomatic treatment all the pneumonia lesions showed improvement. Only in one infant with suspected COVID-19 infection (Case No.18) did we find that the densities of previous existing lung lesions increased with interlobular septal thickening before discharge. This suggests that interstitial fibrosis had developed and whether it will affect the neonate’s pulmonary function in the long term is currently unknown. (Please see Page 16, Line 337-340)

In addition, as mentioned above, among those 12 neonates without COVID-19 had pneumonia-like damage, only one had occasional cough and others were all symptoms-free. As such, in the discharge diagnoses these children were not diagnosed with pneumonia.

*8. Conclusions - clear*

***Reviewer #4:*** *The paper describes infants of 29 mothers with Covid-19. Five infants had Covid-19 (2 confirmed and 3 suspected cases). Detailed description is provided with respect to infants' symptoms, laboratory measures, and x-ray/CT images. Maternal pregnancy complications and delivery are also described. I have relatively minor comments.*

1. *Abstract: Line 74. A retrospective cohort study is an analytical epidemiological study that includes a comparison group (exposed vs unexposed). I suggest this study is a descriptive follow-up study.*

**Response:** We have modified the title as requested by the editor.

(Please see Page 1, Line 1)

2. *Abstract: Can you, please, indicate how many hospitalized infants were hospitalized because they needed hospitalization and how many just for the quarantine. Would these infants be otherwise discharged to home if parents did not opt for the quarantine in the hospital?*

**Response:** Five neonates were hospitalized because they had clinical symptoms warranting observation (one with fever, one with occasional cough, one with birth asphyxia, one with vomiting and bloating, one with pneumonia chest CT images immediately after birth). Thirteen neonates were hospitalized follow parental request. (Please see Page 4, Line 84-85) If parents did not opt for the hospital quarantine, the neonates were discharged home. Given that our hospital was the only designated children’s hospital in Wuhan, if the neonates developed any symptoms at home they would have been admitted back to the hospital.

3. *It would be nice to define the diagnostic criteria for Covid-19 in mothers and in infants (expert consensus) in the Introduction or Methods (the referenced literature may not be easily accessible to all). A brief description of typical signs of Covid-19 pneumonia and how it differs from other pneumonias in newborns would be also appreciated by general medical audience.*

**Response:** Thank you for your suggestion. Due to page-length limitations, diagnostic criteria for COVID-19 among mothers and their infants are listed in the Supporting information. (Please see S1 Text Analysis Plan 2.1 and 2.4 sections)

Due to normal neonatal characteristics (non-specific symptoms and signs, and changed white blood cells and lymphocytes count), it is difficult to identify COVID-19 infection. In this study, chest CT image was determined to be the most powerful diagnostic tool. The typical radiographic findings of COVID-19 pneumonia in our five infants were predominately manifested as focal or diffuse ground-glass opacities located in the peripheral regions, which were distinguishable from bacterial infection but non-specific from other virus pneumonia. (Please see Page 16, Line 330-333)

However, on a global scale, a chest CT scan of all neonates delivered by COVID-19 mothers is unrealistic. Therefore, the accuracy and sensitivity of nucleic acid detection and antibody detection should be improved.

4. *Results Line 193-197: there were 29 women with Covid-19, 16 were diagnosed only by chest CT. Covid-19 symptoms were present only among 14 women. It seems that chest CT was the only diagnostic sign of Covid-19 in some mothers in this study. Is it a reliable?*

**Response:** As mentioned in the S1 Text Analysis Plan, at the beginning of the COVID-19 outbreak in Wuhan there was a shortage of PCR kits so many women could not have nucleic acid testing. Further, the PCR kits had 30% false-negative rate and there was also a problem of sampling. Therefore, among these women the typical viral pneumonia manifestations of a chest CT was considered to be very important for diagnosis. Combined with epidemiological data, it is reasonable to believe that these women with actual pneumonia lesions were infected with COVID-19. (Please see S1 Text Analysis Plan 2.1)

5. *Can you, please, describe the NEC cases in more detail in the results section? Specifically, their gestational age at birth and how many days from birth (to NEC diagnosis)?*

**Response:** Thank you for your suggestion. We have added a brief description of the three neonates with NEC to the results section.

(Please see Page 13, Line 270-274)

6. *Can you please add gestational age at delivery to Table 2 (I suggest in categories, e.g., 20-33, 34-36, 37-38, 39-41, 42+ weeks)?*

**Response:** Thank you for your suggestion. We have added gestational age at delivery to Table 2 and they are now categorized by 35-36, 37-38, 39-41 weeks.

(Please see Page 33, Table 2)

*Minor comments:*

*Line 174: Please use the reference only or specify by the last name, eg "… following the paper by Nissen..."*

**Response:** We have corrected this.

(Please see Page 9, Line 187)
