# Supplementary material for: Neonatal outcome in 29 pregnant women with COVID-19: A retrospective study in Wuhan, China
Source: PLoS Med. 2020 Jul 28;17(7):e1003195. doi: 10.1371/journal.pmed.1003195 (PMC7386573; doi:10.1371/journal.pmed.1003195)
Supplement: Attachment — Submitted filename: Response to Editors.docx [file pmed.1003195.s008.docx]

**New comments from Editors (June 18, 2020 updated):**

*1. Please ensure that the CRediT author contributions listed for each co-author are completed in full. To qualify for authorship, each author must meet at least one of the seven core contributions (conceptualization; data curation; formal analysis; investigation; methodology; software; validation), as well as at least one of the writing contributions (original draft preparation; review and editing). Authors may also be noted against any of the remaining contributions, however these alone will not qualify a contributor for authorship.*

*Please ensure that the full contributions of each author are acknowledged in the "Add/Edit/Remove Authors" section of our submission form, and that all authors meet the criteria for authorship as described above. If they do not meet criteria for authorship please let us know as soon as possible by emailing plosmedicine@plos.org.*

**Response:** I have confirmed the contributions of all authors in the submission system. Dr Yan-Fen Chen, Bi-Heng Cheng, Wen Yang and Yang Chen were the obstetricians, pediatricians, ICU and NICU doctors who took responsibility for collecting and collating the clinical data. Therefore, I’m sure they meet the author’s requirements (as Data curation and Resources).

*2. Thank you for updating the data files. Some of the information on the pregnant women (age, occupation, delivery date) could be used to identify the individuals. Please either redact identifying information or substitute ranges (e.g., 25-30 years old) in the publicly available dataset.*

**Response:** Thank you for your suggestion.

On “age”, we have followed your suggestion that the specific ages were replaced by ranges, including: 20-25, 26-30, 31-35, 36-40 years old.

On “occupation”, in order to compare whether hospital staff have some significant characteristics, we divided occupation into three categories: Employed but not at a hospital, Employed specifically at a hospital and Unemployed.

On “delivery date”, replacing the original specific date, we defined each mother's delivery date (corresponding to the birth date of the neonates) as Day 1, so as to calculate the date diagnosis, admission, discharge, and examination of the both mothers and neonates (Day X). (Please see Page 14, Line 465 of the **marked up document**).

We have updated the uploaded public data (<http://www.medresman.org.cn/pub/cn/proj/projectshshow.aspx?proj=1810>). And in the Table 3, Table 4, Table 5, S1 Table and S2 Text, we also deleted the date of birth and the date of examination.

*3. Thank you for editing the Author Summary. In the first point, please replace “revealed” with “suggest”.*

**Response:** This has been adjusted.

(Please see Page 3, Line 283 of the **marked up document**)

*4. Point 6 of the Author Summary, “radiological features” instead of “radiological changes”?*

**Response:** This has been adjusted.

(Please see Page 4, Line 303 of the **marked up document**)

*5. First line of the “Strengths and limitations” section: “to *integrate*”*

**Response:** This has been corrected.

(Please see Page 26, Line 605 of the **marked up document**)

*6. Please confirm that the text in the upper right corner of the X-ray images in Figures S1 and S2 does not include any identifying information. If there is any identifying information, please edit the image to redact, and note that you have done so in the figure legend.*

**Response:** The text in the upper right corner of the X-ray images is the name of Wuhan Children’s Hospital and the date of examination, which does not contain the patient’s identifying information. But considering that we have deleted all the specific date, we have also hidden the text here. And we have added a statement in the figure legend of Fig 2, S1 Fig and S2 Fig: All images have been de-identified to protect patient privacy.

(Please see Page 18, Line 507-508; Page 41, Line 1283-1284 and 1294 of the **marked up document**; Fig 2, S1 Fig and S2 Fig)

**Comments from Editors (June 5):**

1- *Thank you for providing text excerpts for the STROBE checklist items. Please either provide the full excerpt (no “…”) or describe the section and paragraph where the item appears in the main text (e.g., “Methods, paragraph 1”).*

**Response:** Thank you for your suggestion. A new STROBE statement has been uploaded.

(Please see S1 STROBE Checklist)

2- *Thank you for providing information on how to find the dataset. However, the file (listed as a .xls) did not open on my computer. Please make sure the format is correct.*

**Response:** It may have occurred due to a format conversion problem with the management platform. As such, we have now uploaded the dataset in .xls and .csv format using the same website.

(Please see website http://www.medresman.org.cn/pub/cn/proj/projectshshow.aspx?proj=1810)

3- *Please add "in Wuhan, China" to the title.*

**Response:** We have modified the title as requested and now reads: “Neonatal Outcome in 29 Pregnant Women with COVID-19: A Retrospective Study in Wuhan, China”.

(Please see Page 1, Line 1of the **last marked up document**)

4- *Please add summary demographic details for the mothers to the abstract.*

**Response:** This has been adjusted. The new section reads as follows: “Among 29 pregnant COVID-19-infected women (13 confirmed and 16 clinical diagnosed), the majority had higher education (56.6%), half were employed (51.7%) and their mean age was 29 years. Fourteen women experienced mild symptoms including fever (8), cough (9), shortness of breath (3), diarrhea (2), vomiting (1), and 15 were symptom-free. Eleven of 29 women had pregnancy complications, and 27 elected to have a cesarean section delivery.”

(Please see Page 2-3, Line 231-242)

5- *Abstract, line 113 (of the marked up document): please edit to: “Neonates were hospitalized if they *had* signs *of COVID-19* (5 cases) or their guardians agreed *to* a hospitalized quarantine…” Please confirm whether “signs” or “symptoms” is more accurate in this sentence.*

**Response:** This has been adjusted. The new sentence reads as follows: “Neonates were hospitalized if they had symptoms (5 cases) or their guardians agreed to a hospitalized quarantine (13 cases)”.

(Please see Page 2, Line 225-226)

6- *Please edit the Abstract Conclusions. While we appreciate providing a specific response here, the figures presented are better suited to the Abstract Methods and Findings. A more appropriate Conclusion could be: In this study, we observed that COVID-19 or radiological features of pneumonia in some, but not all, neonates born to women with COVID-19. These findings suggest that intrauterine or intrapartum transmission is possible.”*

**Response:** Thank you for your suggestion – the modification has been made.

(Please see Page 3, Line 252-255)

7- *Thank you for providing an Author Summary. However, this should not simply restate the information in the Abstract. Please edit the “What did the researchers do and find?” and “What do these findings mean?” to provide a non-technical summary of the findings and implications. Please also limit the sections to 2-3 bullet points each.*

**Response:** Thank you for your suggestion – the modification has been made.

(Please see Page 4, Line 269-285)

8- *Additionally, please adjust the “What did the researchers do and find” section to reflect the reviewer comments about the neonatal pneumonia findings which were not conclusive and could be a little misleading.*

**Response:** This has been adjusted. This section reads as follows: “•	Among the 30 neonates born to 29 pregnant COVID-19-infected women (one set of twins), five were diagnosed as having COVID-19 (two confirmed and three suspected) and other 12 neonates presented radiological features of pneumonia but none were diagnosed with pneumonia.” And “•	A considerable number, but not all, neonates born to COVID-19-infected women developed COVID-19 (5/30) or had pneumonia-like radiological changes (17/30).”

(Please see Page 4, Line 273-276, 280-281)

9- *Introduction, line 178: more accurate to say “...of all women with *confirmed* COVID-19 infections…”?*

**Response:** This has been adjusted as requested and the new sentence now reads as follows: “...of all women with confirmed or clinical diagnosed COVID-19 infections…”.

(Please see Page 5, Line 312)

10- *Introduction line 180: as this is not a randomized trial, please refrain from using causal language (eg “effect”). Instead please change to “... to assess neonatal outcomes associated with maternal COVID-19 infection.”*

**Response:** Thank you for your suggestion – the modification has been made.

(Please see Page 6, Line 317)

11- *At line 210, we suggest adapting the text to "For this retrospective study in clinical practice, the requirement for written informed consent was waived by the ethics committee.".*

**Response:** Thank you for your suggestion – the modification has been made.

(Please see Page 6, Line 334-335)

12- *In the results section of your main text, please state the breakdown of the neonates by sex (we may have missed this in the text or tables).*

**Response:** We have recorded the sex of each hospitalized neonate in Table 4 and Table 5. In the Results section, we have added sex description to some important neonate groups, including five neonates with COVID-19 and 13 other hospitalized neonates. Further, we have added the sex distribution of each group to the flow chart.

(Please see Page 6, Line 440; Page 18, Line 490-491, 493; Table 4 and Table 5; Fig 1)

13- *At line 387, please make that "had contact".*

**Response:** This has been corrected.

(Please see Page 25, Line 538)

14- *At line 388, please make that "... which suggests vertical and intrapartum transmission." or similar.*

**Response:** This has been adjusted.

(Please see Page 25, Line 539)

15- *At line 439, please avoid claiming "the first", for example, and where necessary please amend the text to "to our knowledge the first" or similar. E.g, “This is the first study to our knowledge to explore the association between maternal COVID-19 infection and neonatal COVID-19 infection or other health conditions.”*

**Response:** Thank you for your suggestion – the modification has been made.

(Please see Page 26, Line 582-584)

16- *At lines 107 & 481, please make that "COVID-19-infected women/neonates" or similar.*

**Response:** Thank you for your suggestion – the modification has been made.

(Please see Page 2, Line 257; Page 4, Line 293, 299, 306, 310; Page 9, Line 431; Page 14, Line 468; Page 25, Line 573; Page 26, Line 594; Page 28, Line 654; Page 31, Line 741, 746)

17- *Discussion, line 440: please change to: “All mothers with confirmed COVID-19 infections who gave birth…”. Please also provide a more specific time frame than “around February 2020”, and specify this includes all mothers who gave birth at a hospital in China, allowing for the possibility of births that were not identified and included in this study.*

**Response:** We have modified the text as follows: “All women with confirmed or clinical diagnosed COVID-19 infections who gave birth at two of the five designated general hospitals between January 30 to March 10, 2020 during the most severe time of the epidemic in China were included in this study.”.

(Please see Page 26, Line 610-613)

18- *Line 469: more accurate to say “...limited statistical power.”?*

**Response:** Thank you for your suggestion – the modification has been made.

(Please see Page 26, Line 636)

19- *Line 472: “Our findings suggest…”*

**Response:** Thank you for your suggestion – the modification has been made.

(Please see Page 26, Line 638)

20- *Line 481: “In *the* five COVID-19 neonates…”*

**Response:** Thank you for your suggestion – the modification has been made.

(Please see Page 27, Line 654)

21- *Please incorporate your response to reviewer 2 into the second section of the Discussion, citing the most current studies on pregnant women with COVID-19 and their children, and placing your findings in the context of these other studies.*

**Response:** Thank you for your suggestion. We have added the current studies on pregnant women with COVID-19 infections and their children, and placed our findings in the context of these studies in the discussion section. While about the response to reviewer 2, we have added it to the Discussion-Strengths and limitations section.

(Please see Page 25, Line 568-578; Page 26, Line 608, 611-612; Page 27, Line 625)

22- *In the Discussion, please discuss the limitations in a bit more depth, particularly those around the radiological findings among neonates and the diagnosis of some mothers without PCR testing.*

**Response:** For the radiological findings among neonates, we primarily discussed this in paragraphs 2-3 in Discussion-Research implications section. The main limitation is that 12 hospitalized neonates did not have fever or other symptoms, so they did not retain tracheal aspirate to detect other pathogens. This made it difficult to diagnose what kind of pneumonia these neonates had. Further, for the clinical diagnosed women without PCR testing, a study included 1014 patients in Wuhan who underwent both chest CT and RT-PCR tests. And they found “the sensitivity of chest CT in suggesting COVID-19 was 97% (95%CI, 95-98%, 580/601 patients) based on positive RT-PCR results.” Therefore, the clinically diagnosed women were considered to be COVID-19 infected. We have added these two points to the limitations section.

(Reference: Ai T, Yang Z, Hou H, Zhan C, Chen C, Lv W, et al. Correlation of Chest CT and RT-PCR Testing in Coronavirus Disease 2019 (COVID-19) in China: A Report of 1014 Cases. Radiology. 2020;200642. pmid: 32101510)

(Please see Page 27, Line 628-634)

23- *S1 and S2 Table seem to be missing, please upload. Page limits should not be an issue, S1 Table can be added to the main text as Table 5.*

**Response:** We have re-uploaded the supporting information and the original S1 Table was added to the main text as Table 5. The original S2 Table was renamed as S1 table.

(Please see Page 20-23)

24- *Please present all new results in the Results section. The section beginning at line 507 of the Discussion seems to present new information on the follow-up of several neonates that should be in the Results section.*

**Response:** We have added this information to the Results section as requested.

(Please see Page 24, Line 540-545)

25- *Rather than using “Case X”, please refer to “patients” or “individuals”.*

**Response:** Thank you for your suggestion. We have modified all the “Case No.X” into “Patient X”.

26- *Line 359: PLOS does not allow for data that is “not shown”. Please add this information to the tables.*

**Response:** We have added the symptoms of the three NEC neonates to Table 5 in the “Other symptoms” part (Please see Table 5). A new figure of abdominal X-ray images of the three NEC neonates was also added (S2 Fig). The paragraph was modified as follows: “These neonates (Patient 2, 14, 15) developed NEC after birth at 37^+6^, 40^+1^, 37^+2^ weeks’ gestation, respectively (Table 5). The first symptoms included bloody stools (Patient 2 and Patient 14 both at Day 4), repeated vomiting and bloating (Patient 15 at Day 2). Further, abdominal X-rays showed abnormalities including intermediate abdominal pneumatosis, and indistinct intestinal space (S2 Fig).”.

(Please see Page 24-25, Line 548-558; Page 20-23, Table 5; S2 Fig)

27- *Please remove the information on conflicts of interest, funding and data availability from the end of the main text. This information will appear in the metadata in the event of publication (via the submission form).*

**Response:** We have removed these sections.

**Format modification:**

*1. FIGURES and GRAPHICS:*

*1. Please ensure that all main figure files are cited in ascending numerical order in the main text of the article.*

**Response:** Yes, all main figure files are cited in ascending numerical order.

*2. Please ensure that all main figures are referenced in the manuscript as Fig 1, Fig 2, etc. (including capitalization), rather than Figure 1, Figure 2, etc. Please note, however, that the file names themselves must not include the space, i.e. Fig1.tif, Fig2.tif, etc.*

**Response:** This has been adjusted.

*3. Please ensure that all main figure legends are placed after the paragraph containing their first in-text citation.*

**Response:** This has been adjusted.

*TABLES:*

*1. Please ensure that all main tables in their entirety (including titles and footnotes), are placed after the paragraph containing their first in-text citation.*

**Response:** This has been adjusted. Table 5 was placed horizontally due to the large number of cases covered.

*METADATA:*

*1. Please ensure that the CRediT author contributions listed for each co-author are completed in full. To qualify for authorship, each author must meet at least one of the seven core contributions (conceptualization; data curation; formal analysis; investigation; methodology; software; validation), as well as at least one of the writing contributions (original draft preparation; review and editing). Authors may also be noted against any of the remaining contributions, however these alone will not qualify a contributor for authorship.*

*Please ensure that the full contributions of each author are acknowledged in the "Add/Edit/Remove Authors" section of our submission form, and that all authors meet the criteria for authorship as described above. If they do not meet criteria for authorship please let us know as soon as possible by emailing* [*plosmedicine@plos.org*](mailto:plosmedicine@plos.org)*.*

**Response:** All authors make important contributions to the article.

*2. Please ensure only the authors' initials are included in the competing interest statement, not their full names or titles.*

**Response:** This has been adjusted in the submission form.

*3. In accordance with PLOS' data policy, please ensure that your Data Availability Statement in the submission form clearly identifies how readers can access your data. Data cannot be available on request, or only accessible by contacting one of the authors. Note that your Data Availability Statement will be typeset as it is written, so please ensure it is in complete sentences and appears as you would like it to in the published version.*

*For details on our data policy, please see http://journals.plos.org/plosmedicine/s/data-availability*

**Response:** This has been adjusted in the submission form.

*4. You have added Andrew Kawai as an author since the last revision. PLOS follows the Committee on Publication Ethics (COPE: https://publicationethics.org/) guidelines for the addition/removal of authors. We therefore require that any changes to the author listing on a manuscript are confirmed and agreed by all authors, including the authors being removed or added. Please could you contact all authors - including the authors being removed/added - to obtain their emailed confirmation that they agree to this change, and then forward all these confirmations to us in one PDF file?*

*Please note that all authors must fulfill the ICMJE criteria for authorship. Seehttp://journals.plos.org/plosmedicine/s/authorship for details.*

**Response:** All authors have written to agree the addition of Andrew Kawai's name to the author list. I have sent the author's confirmation letter to PLOS Medicine’s email (plosmedicine@plos.org).

*ARTICLE FILE:*

1. *Please indicate the corresponding author in the byline by placing an asterisk (*) after their affiliation number.*

**Response:** This has been adjusted.

2. *Please include the corresponding author's email address on the title page of your manuscript, indicated by an asterisk (*). Only an asterisk and the email address itself are required.*

**Response:** This has been adjusted.

3. *The name for your co-author Ben W. Mol does not match across the article file and the submission form. Please ensure that all author names match verbatim, including the use of a middle name/initials and any special characters (accents, umlauts, etc.), as correct spelling and formatting of author names is vital for accurate indexing in PubMed.*

**Response:** This has been adjusted and now reads: Ben Willem Mol. And we also adjusted it in the submission form.

4. *Please remove the funding information from your manuscript file. The published version is pulled automatically from the “Financial Disclosure” field in the submission form and must not be duplicated. Please therefore take this opportunity to check that the information in this field is correct and completed in full.*

**Response:** We have removed the section.

5. *Please remove the Competing Interests section from your manuscript file. The published version is pulled automatically from the "Competing Interests" field in the submission form and must not be duplicated. Please therefore take this opportunity to check that the information in this field is correct and completed in full.*

**Response:** We have removed the section.

6. *Please remove your Author Contributions section from your manuscript file as this information is automatically pulled from the authors section of our submission form and must not be duplicated. Please therefore take this opportunity to check that the information in this field is correct and completed in full.*

**Response:** We have removed the section.

7. *Please remove the data availability information from your manuscript file as this is automatically pulled from the "Data Availability" field in the submission form and must not be duplicated. Please therefore take this opportunity to check that the information in this field is correct and completed in full.*

**Response:** We have removed the section.

8. *Please ensure that your Ethics statement is available in the Methods section of your manuscript in its entirety.*

**Response:** We have confirmed it.

*REFERENCES:*

1. *Please ensure your references are in the style of PLOS Medicine (ICMJE/Vancouver style). The reference style guide can be found here:http://journals.plos.org/plosmedicine/s/submission-guidelines#loc-references. You can also find a downloadable EndNote file in these guidelines to assist you. Please do a manual check as well, as the EndNote file does not catch everything. Please note that the references will be typeset as they appear so please ensure the format is consistent.*

**Response:** This has been adjusted.

2. *Please put all reference citations in square brackets separated by either commas or dashes with no spaces, e.g. [1,2,3] or [1-3].*

**Response:** This has been adjusted.

3. *Please make sure all references are cited in ascending numerical order in the text.*

**Response:** We have confirmed it.

*SUPPORTING INFORMATION:*

1. *Please ensure that all Supporting Information files use the following format verbatim throughout the manuscript (including legends and all in-text citations): S1 Fig, S1 Table, S1 Text, S1 Data, etc. Each file type should start at 1 and increase numerically. Please note, however, that the file names themselves must use an underscore rather than a space, i.e. S1_Fig.tif, S1_Table.xlsx, S1_Text.doc, S1_Data.xlsx, S1_PRISMA_Checklist.docx, etc.*

**Response:** This has been adjusted.

2. *Please ensure that each supporting information file has a legend at the end of the manuscript file, after the Reference list.*

**Response:** This has been added.

3. *Please note that your submission cannot contain any references to generic "supporting information" files and instead must refer to specific files using our naming convention (S1 Fig, S1 Table, S1 Text, S1 Data, S1 PRISMA Checklist, etc.)*

**Response:** We have confirmed it.
